# Wearable and Implantable Devices for Continuous Monitoring of Muscle Physiological Activity: A Review

**DOI:** 10.1002/advs.202509934

**Published:** 2025-09-25

**Authors:** Zhengwei Liao, Ata Golparvar, Mohammad Javad Bathaei, Filipe Arroyo Cardoso, Clementine M. Boutry

**Affiliations:** ^1^ Department of Microelectronics Delft University of Technology Delft 2628 The Netherlands

**Keywords:** bioelectronics, biomechanics, electrophysiology, soft and flexible electronics, tissue oxygenation

## Abstract

Muscle plays a vital role in movement and metabolic regulation, establishing it as a cornerstone of overall health. Monitoring muscular parameters is critical for disease diagnosis, post‐surgical recovery, and human–machine interface control. In recent decades, numerous technologies have emerged to monitor muscular biophysical and biochemical processes. The field has transitioned significantly from reliance on large, clinic‐bound instrumentation to the development of miniaturized wearable and implantable systems capable of continuous real‐time monitoring in everyday settings. This article presents a critical overview of recent advances, with a focus on material and device innovations in muscular monitoring. Starting with the fundamental characteristics of muscle tissue and the physiological origins of biosignals, the discussion subsequently shifts to recent developments in wearable and implantable bioelectronic systems tailored to monitor electrophysiological, biomechanical, and tissue oxygenation signals. Finally, current research challenges and outline emerging opportunities are highlighted in muscular monitoring. Owing to its interdisciplinary nature and growing societal demand for personalized healthcare, muscular monitoring is poised to catalyze transformative innovations in both clinical and consumer applications.

## Graphical abstract

Recent advances in materials and device engineering enable continuous, real‐time monitoring of muscle activity via wearable and implantable systems. This review critically summarizes emerging technologies for tracking electrophysiological, biomechanical, and oxygenation signals, outlines fundamental principles, and highlights key challenges and opportunities that define the future landscape of personalized muscular health monitoring.

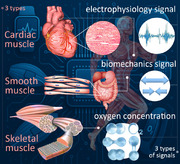

## 1 Introduction

Muscles are essential for movement, circulation, and organ function, serving as the body's primary contractile tissues. Muscles are classified into three types: skeletal, cardiac, and smooth muscles. Skeletal muscles enable voluntary movement and posture; cardiac muscles drive the rhythmic pumping of the heart; and smooth muscles regulate the dimensions and motility of internal organs such as the stomach and intestines. Together, these muscles are essential for maintaining physiological function and homeostasis. They generate a range of biosignals, including electrophysiological, biomechanical, and oxygenation‐related, that reflect neural innervation, contractile activity, and metabolic processes. Monitoring these signals offers valuable insights into muscle health, performance, and adaptation.^[^ ^1^, ^2^, ^3^ ^]^ The biofeedback derived from muscle monitoring is critical for medical diagnostics, the therapeutics of neuromuscular disorders, rehabilitation, human–machine interface control, and human augmentation for virtual and augmented reality.^[^ ^4^, ^5^, ^6^ ^]^

Continuous monitoring of muscle functions is an emerging field that has attracted significant research interest across multiple scientific disciplines.^[^ ^7^, ^8^ ^]^ Based on the type of biosignals detected, muscle tracking technologies are generally classified into three categories: electrophysiology, biomechanics, and oxygenation. Electrophysiological monitoring involves tracking action potentials in muscle tissue, which trigger muscle contraction. In cardiac and skeletal muscles, these electrical signals can be recorded using electrocardiography (ECG) and electromyography (EMG), respectively. The electrophysiological signals can also be captured by detecting the magnetic fields generated by current flowing through a nerve, a technique known as Magnetomyography (MMG). Muscular biomechanics, derived from the contraction and relaxation of muscle fibers, drive the movement of body segments and internal organs. Techniques such as force myography (FMG) and electrical impedance myography (EIM) monitor biomechanical activity by detecting volumetric changes in muscle tissue. Other techniques, Acoustic myography (AMG) and sono‐myography (SMG), analyze muscle vibrations and their interaction with acoustic waves. In addition to electrophysiology and biomechanics, muscles require adequate oxygenation to support aerobic metabolism. Photoplethysmography (PPG), electrochemical biosensors, and luminescence‐based sensing platforms are among the most widely used methods for assessing tissue oxygen concentration in tissue. The first muscle monitoring instruments were wired, bulky, and primarily used for clinical diagnostics and vital signs monitoring. In recent years, advances in microengineering and material science have driven a shift toward miniaturized, wireless wearable and implantable devices. These compact systems aim to minimize interference with physical activity, allowing their usage in ambulatory or home environments.^[^ ^9^, ^10^, ^11^ ^]^ Meanwhile, rising public interest in daily health management has stimulated the growth of proactive healthcare solutions and accelerated the adoption of remote, real‐time monitoring technologies.^[^ ^12^ ^]^

Several review papers have addressed specific muscular monitoring technologies. Particularly, Tchantchane et al. and Putcha et al. reviewed muscle monitoring technologies, focusing primarily on signal processing and application scenarios.^[^ ^13^, ^14^ ^]^ However, their work did not cover sensing modalities comprehensively. Wang et al. systematically summarized myography techniques but limited their scope to wearable systems for high‐resolution muscle‐machine interfacing and overlooked implantable approaches.^[^ ^15^ ^]^ In this review, we overcome a gap in the literature by analyzing recent advancements in muscular monitoring techniques, with a focus on innovations in materials and device architectures. We begin with a concise overview of muscle biology, emphasizing key structural and signaling mechanisms of skeletal, cardiac, and smooth muscles. We then examine state‐of‐the‐art methods for monitoring muscle electrophysiology, biomechanics, and tissue oxygenation, with a focus on technological progress over the past decade. Finally, we discuss current limitations of wearables and implantable muscle monitoring systems and propose future research directions to advance the field.

## 2 Human Muscles: From Anatomy to Function and Signals Generation

### 2.1 Skeletal, Cardiac, and Smooth Muscles

**Figure 1:**
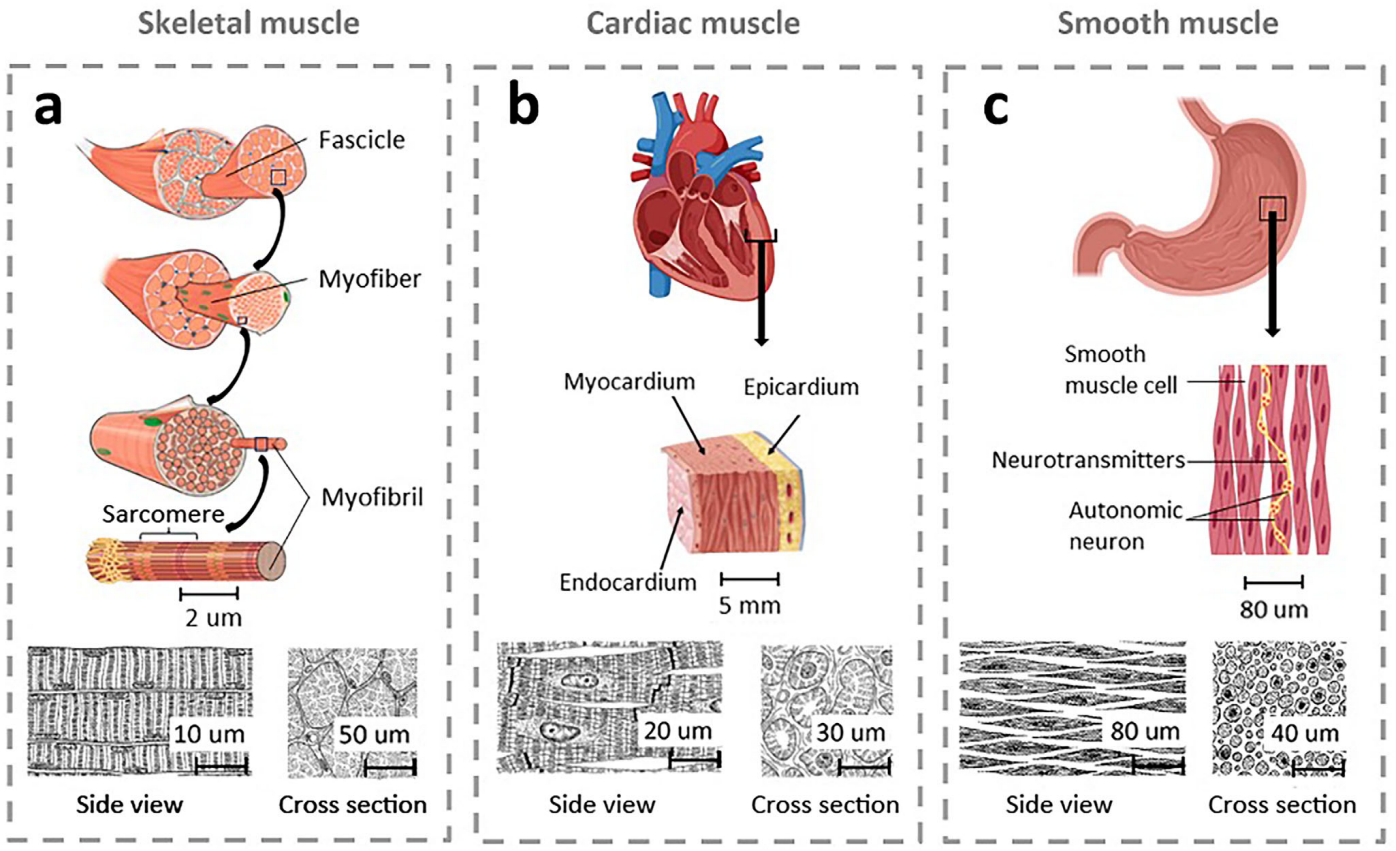
Hierarchical structures (top) and microscope images (bottom) depicting the three types of muscle in the human body: a) skeletal,^[^ ^14^, ^19^, ^25^ ^]^ b) cardiac,^[^ ^19^, ^25^ ^]^ and c) smooth muscles.^[^ ^19^ ^]^ All pictures are reproduced with permission.

Muscle tissue in homo sapiens is classified into three types: skeletal, cardiac, and smooth muscles.^[^ ^16^ ^]^ Unique structures and specialized innervation characterize each type. Skeletal muscles attach to bones via tendons at both ends and represent ≈40% of the body mass. They are responsible for voluntary body movements and are ultimately controlled by the central nervous system through the peripheral nervous system.^[^ ^17^ ^]^ A skeletal muscle contains several fascicles, which in turn are composed of a group of myofibers (**Figure** **1**a). Myofibers are elongated cells with multiple nuclei that contain myofibrils. The myofibrils are thread‐like structures made up of repeating units called sarcomeres, which are the basic contractile units of the muscle.^[^ ^14^, ^18^ ^]^ Myofibrils consist of actin (thin) and myosin (thick) filaments,^[^ ^19^ ^]^ which slide past each other during contraction, shortening the muscle fiber and generating force.

Figure 1b shows the structure of cardiac muscles, which compose the heart walls. Cardiac muscles are structured into three layers: epi‐, myo‐, and endo‐cardium. The innermost layer, the endocardium, separates the myocardium from the blood within the heart chambers. The myocardium, composed of cardiomyocytes and fibroblasts, enables the heart's pumping function and is regulated by the autonomic nervous system through involuntary cardiomyocyte contractions. Unlike skeletal and smooth muscle cells, cardiac muscle cells are uninucleate, interconnected by intercalated discs that facilitate synchronized mechanical and electrical coupling.^[^ ^20^ ^]^ Lastly, the tough and fibrous epicardium provides a protective outer layer.^[^ ^21^, ^22^ ^]^

Figure 1c illustrates smooth muscles characterized by their lack of striations. Smooth muscles contain spindle‐shaped cells, which typically have a single nucleus. An autonomic nervous system innervates the smooth muscle, providing contractile ability to hollow organs and tubes such as the urinary bladder, ureters, intestines, and blood vessels.^[^ ^23^ ^]^ Although smooth muscle contraction and relaxation are slower than other muscle types, they can sustain contractions for extended periods without fatigue. Unlike skeletal and cardiac muscles, smooth muscles can contract in response to neurotransmitters without requiring action potentials.^[^ ^24^ ^]^

### 2.2 Muscles Function, Signals Generation, and Regulation

In skeletal and cardiac muscles, the nervous system regulates muscle activity (i.e., contraction and relaxation) by generating and propagating action potentials within the muscle cells. These neural signals can be recorded at the tissue and organ level. At the skin level, electrophysiological signals are acquired by surface electrodes that detect voltage fluctuations from underlying action potentials. The skin‐electrode interface is typically modeled as a resistor‐capacitor (RC) circuit, where the capacitance arises from the insulating stratum corneum and the resistance reflects ionic conduction through sweat and deeper skin layers. Electrical signaling in muscle cells results from ion movement across membranes, where differential permeabilities of K⁺, Na⁺, Ca^2^⁺, and Cl^−^ generate concentration gradients and membrane potentials of tens of millivolts.^[^ ^26^, ^27^, ^28^ ^]^ Muscle cells cycle through resting, depolarization, and repolarization states. Ion pumps maintain the resting potential, while sufficient stimulation triggers depolarization and an action potential, leading to contraction. Repolarization restores the resting state, allowing relaxation.^[^ ^29^, ^30^ ^]^ In both skeletal and cardiac muscles, action potential triggers muscle contraction via excitation‐contraction coupling, linking electrical excitation to muscle activity. Action potential propagation prompts Ca^2+^ influx, releasing stored Ca^2+^ from the sarcoplasmic reticulum. These Ca^2+^ ions bind to sarcomeres, initiating cross‐bridge formation between actin and myosin with adequate Adenosine Triphosphate (ATP) supply.^[^ ^31^, ^32^ ^]^

For skeletal muscles, the stimulus originates from the primary motor cortex and travels through the spinal cord to the motor unit. Each motor unit comprises a motor neuron and multiple skeletal muscle fibers interconnected at the neuromuscular junction. Upon the arrival of the nerve impulse at the neuromuscular junction, neurotransmitters are released from vesicles and bind to receptors on the muscle fiber membrane.^[^ ^33^, ^34^ ^]^ This biochemical interaction triggers an action potential that spreads along the muscle, resulting in the contraction of the corresponding skeletal muscle cells. According to Henneman's size principle, motor units are recruited in an orderly sequence from the smallest, low‐threshold units to progressively larger, high‐threshold units as greater force is required.^[^ ^35^ ^]^ Small motor units, typically involved in fine motor control, innervate fewer muscle fibers and thus generate more distinct and cleaner signals. Larger units, recruited during stronger contractions, activate more muscle fibers simultaneously, producing higher‐amplitude and more complex waveforms due to signal summation.^[^ ^36^ ^]^ In cardiac muscles, the autonomic nervous system exerts involuntary control of the heart‐pumping activity. The interplay between the sympathetic and parasympathetic nervous systems regulates the heart's activity.^[^ ^37^, ^38^ ^]^ The cardiac conduction system, beginning at the sinoatrial (SA) node, transmits action potentials through the heart via the internodal pathways, atrioventricular (AV) node, bundle of His, bundle branches, and Purkinje fibers, ensuring coordinated atrial and ventricular contractions.^[^ ^39^ ^]^ Cardiac and skeletal muscles primarily use aerobic metabolism for energy. Under intense exercise or insufficient oxygen supply, these muscles supplement their ATP needs through anaerobic metabolism.^[^ ^40^ ^]^ In this pathway, glucose is reduced to lactate, producing hydrogen ions and phosphate that lower intracellular pH, inhibiting metabolic enzymes, hallmarks of muscle fatigue.^[^ ^41^ ^]^ Aerobic metabolism produces ATP through four steps: substrate uptake, glycolysis, mitochondrial oxidation of acetyl‐CoA via the Krebs cycle, and the Electron Transport Chain. These reactions produce ATP, CO_2_, and H_2_O, essential for sarcomere cross‐bridge formation and muscle contraction.^[^ ^42^ ^]^ Therefore, maintaining a sufficiently high oxygen concentration is crucial for muscle cells to convert chemical energy into mechanical energy, supporting daily muscle activities effectively.

## 3 Electrophysiological Signals Monitoring

Electrophysiological monitoring systems are employed in early disease diagnostics and clinical procedures, while also enabling remote monitoring and supporting advanced human–machine interfaces by providing real‐time biofeedback.^[^ ^43^, ^44^, ^45^, ^46^, ^47^, ^48^ ^]^ Electrophysiological signals, such as cardiac (electrocardiography, ECG), skeletal muscle (electromyography, EMG), ocular (electrooculography, EOG), neural (electroencephalography, EEG; electrocorticography, ECoG), and gastrointestinal (electrogastrography, EGG), propagate throughout the body and can be recorded to evaluate various physiological functions.^[^ ^43^, ^49^, ^50^ ^]^ For muscle vitality assessment, ECG monitors cardiac muscle activity, whereas EMG technology focuses on skeletal muscle contractions. This section reviews recent advances in ECG and EMG technologies, focusing on wearable and implantable systems. We also highlight recent progress in magnetomyography (MMG), a complementary technique to EMG that captures the magnetic fields generated by muscle activation.

### 3.1 Biopotentials of Cardiac Muscles: Electrocardiography (ECG)

**Figure 2:**
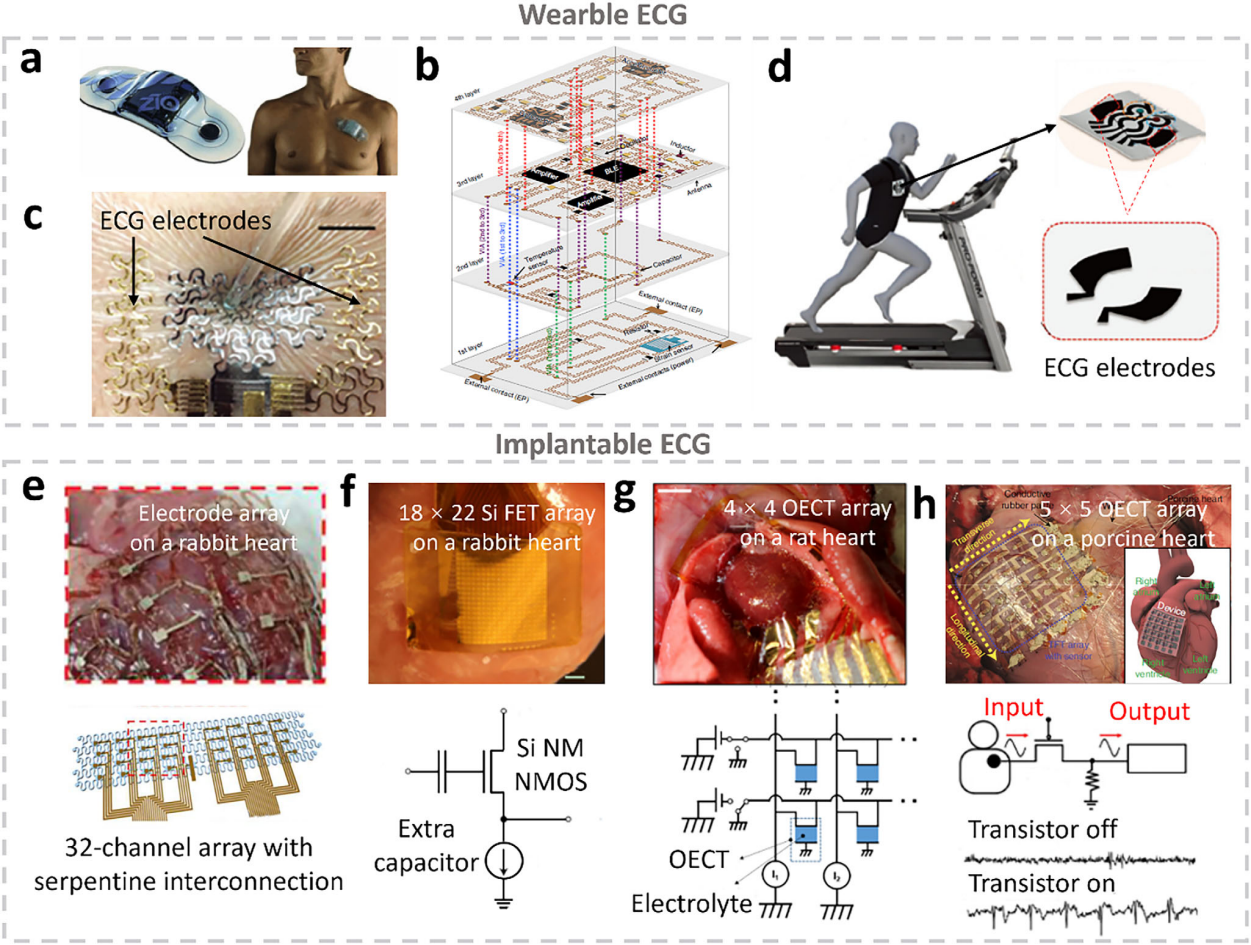
Wearable and intracardiac Electrocardiography (ECG) monitoring systems. a) 14‐day single‐use, ECG monitoring patch, Zio Patch, developed by iRhythm Technologies.^[^ ^60^ ^]^ b) A 3D‐integrated 4‐layer stretchable patch where the wearable ECG electrodes are interfaced with other components, including antenna, amplifier, capacitor, and Bluetooth.^[^ ^85^ ^]^ c) A soft electro‐mechano‐acoustic cardiovascular (EMAC) sensing e‐tattoo, including serpentine Au electrodes on stretchable medical tapes (Tegaderm, 3M) for ECG measurements. Scale bar: 1 cm.^[^ ^91^ ^]^ d) An all‐fibrous nanohybrid multiplexed biosensing patch integrated into the textile. The inset shows the electrodes for ECG monitoring.^[^ ^107^ ^]^ e) Stretchable epicardial 32‐channel electrode array for mapping intracardiac electrograms. The inset image shows the attachment of the array on the in vivo rabbit heart.^[^ ^128^ ^]^ f) A capacitively coupled 18 × 22 array of multiplexed flexible silicon transistors array positioned on a rat heart (top) and the image showing a capacitively coupled transistor design. Scale bar: 3 mm.^[^ ^132^ ^]^ g) A 4 × 4 stretchable organic electrochemical transistor (OECT) array on a honeycomb grid parylene substrate mounted at in vivo rat heart (top) and the circuit diagram of this 4 × 4 stretchable OECT array (bottom). Scale bar: 5 mm.^[^ ^134^ ^]^ h) An epicardial bioelectronic patch where a 5 ×5 rubbery thin‐film transistor (TFT) array is integrated for mapping the electrophysiological activity of a living porcine heart (top) and circuit diagram of a TFT sensing element and the recorded ECG signal when the transistor gate is on and off (bottom).^[^ ^135^ ^]^ All pictures are reproduced with permission.

The ECG signal originates from the action potentials generated by cardiac myocytes, which propagate through the heart's electrical conduction system, including the SA node, AV node, and Purkinje fibers. Although ECG can be measured directly from the endocardium, it is most commonly recorded non‐invasively from the skin. Due to the conductive properties of body tissues such as muscle and fat, cardiac electrical signals reach the skin surface, allowing surface electrodes to effectively capture real‐time data for diagnosing conditions like arrhythmias and myocardial infarction.^[^ ^51^, ^52^ ^]^ The standard 12‐lead ECG provides a 3D spatial assessment with amplitudes typically ranging from 10 µV to 4 mV.^[^ ^53^, ^54^, ^55^ ^]^ It consists of 10 electrodes placed on the chest, arms, and legs to capture ECG from 12 different directions, termed leads.^[^ ^56^ ^]^ Although multi‐lead ECG offers comprehensive diagnostics, single‐ or dual‐lead systems remain valuable; for example, basic arrhythmias can be preliminarily identified using a single ECG lead,^[^ ^57^ ^]^ simplifying testing procedures. Various electrode placements, such as chest, ear, forehead, neck, and single‐arm ECG, have been adapted in wearable devices for continuous monitoring applications.^[^ ^55^ ^]^ Commercial wearable ECG sensors are available for handheld operation (e.g., Apple Watch Series 4 and AliveCor KardiaMobile)^[^ ^58^, ^59^ ^]^ and hands‐free format (e.g., Zio Patch and BioTel Heart MCOT Patch),^[^ ^60^, ^61^ ^]^ capable of recording one, two, six, or more leads, depending on electrode configuration. For instance, the Zio Patch (**Figure** **2**a) enables single‐lead continuous ECG monitoring for up to 14 days.^[^ ^60^ ^]^

As a well‐established pre‐diagnostic tool with a long history of application, ECG has inspired extensive research aimed at developing next‐generation systems that improve signal‐to‐noise ratio (SNR), enable system miniaturization, enhance user comfort during prolonged use, and increase measurement stability, spatial resolution, and accuracy.^[^ ^62^, ^63^, ^64^ ^]^ Conventional clinical ECG systems typically employ pre‐gelled Ag/AgCl wet electrodes to reduce skin‐electrode impedance. However, their tendency to dry out over time makes them unsuitable for long‐term wearable applications, as they compromise signal quality and user comfort.^[^ ^63^ ^]^ To address these limitations, researchers have developed dry electrodes for wearable ECG systems intended for continuous and long‐term monitoring. Dry electrodes establish electrical contact with the skin through conductive materials, eliminating the need for gels and reducing skin irritation.^[^ ^65^, ^66^, ^67^, ^68^, ^69^, ^70^ ^]^ Based on their interface with the skin, wearable ECG systems can be categorized into (1) skin‐interfaced devices such as smart patches and electronic tattoos (e‐tattoos) and (2) smart textiles integrated with electronic or electroconductive fibers (e‐textiles).^[^ ^54^ ^]^

One must carefully balance different material properties to design accurate and reliable wearable ECG electrodes. Soft, breathable substrates are essential for comfort and close‐skin contact, which enhances signal quality. Conductive layers are commonly made from metals, carbon‐based materials, and conductive polymers, chosen for their combined material properties, including mechanical compliance, conductivity, long‐term stability, and biocompatibility.^[^ ^62^ ^]^ To maintain reliable signal acquisition during motion, electrodes must exhibit good conformability to seamlessly adapt to the microtopography of skin. This requires materials with low bending stiffness and a low Young's modulus (typically <100 kPa).^[^ ^71^ ^]^ Additionally, these devices also include structural designs (e.g., ultrathin films, serpentine traces, mesh layouts) that accommodate natural skin movements. Moreover, user comfort over prolonged usage is enhanced by highly breathable designs, which reduce moisture accumulation and minimize skin irritation. Low electrode‐skin impedance is vital to minimize signal distortion and baseline noise, especially in the low‐frequency range. Achieving low impedance requires high‐conductivity materials, optimized surface area, and skin‐compatible coatings to enhance ionic/electronic charge transfer.

Additionally, ongoing research targets improvements in efficient power supplies, data transmission systems, and edge processing units, key components for continuous ambulatory ECG monitoring, although these topics are beyond the scope of this review.^[^ ^72^ ^]^

#### 3.1.1 Smart Electrocardiography (ECG) Patches and Tattoos

ECG patches represent the most common sensing format, utilizing self‐adhesive conductive materials on flexible substrates to achieve strong skin adhesion and conformability to various body contours. Designing optimal electrode structures and selecting appropriate materials, such as carbon‐based materials, metals, and conductive polymers, is crucial for enhancing the sensing performance at the skin‐electrode interface. In addition, soft electrodes incorporating hydrogels or gel‐like matrices are frequently employed in these patches to enhance user comfort and signal quality by improving interface conformity.^[^ ^62^, ^73^ ^]^

Continued advances in materials science are driving the development of next‐generation ECG patches. Shi et al. introduced a unique “living hydrogel”, a dual‐network material composed of gelatin and gelatinized tapioca starch designed to encapsulate the commensal bacterium *Staphylococcus epidermidis*. This living hydrogel functioned as a biocompatible encapsulation layer and biological treatment layer. Utilizing this new material, they developed an active bio‐integrated living electronics platform capable of recording 6‐lead ECG signals together with effective psoriasis treatment.^[^ ^74^ ^]^ Mechanical mismatch between rigid electrodes and soft, stretchable polymers has been a persistent challenge in stretchable conductive nanocomposites for ECG monitoring. To address this, Lu et al. applied a cryogenic transfer approach (−196 °C) to create highly stretchable conductive laser‐induced graphene (LIG)/hydrogel nanocomposites. The flash‐frozen process increased the interfacial binding energy between defective porous graphene and crystallized water within the hydrogel, leading to improved interface adhesion between LIG and hydrogel. The hydrogel also served as the energy dissipation layer, introducing the deflected cracks in LIG, which offered out‐of‐plane electrical pathways and unprecedented 4‐time enhancement of stretchability of the ECG electrodes.^[^ ^56^ ^]^ Another emerging direction in wearable ECG sensing is the transition from passive to active electrodes enabled by transistor technologies. Active electrodes integrate signal amplification and noise reduction via advanced circuit designs and compensation.^[^ ^75^ ^]^ In 2024, Li et al. introduced a hydrogel with semiconducting properties. It was an n‐type water‐soluble semiconducting polymer, P(PyV)‐H, featuring a 3D porous network that facilitated efficient ion and molecule transport. The organic electrochemical transistor (OECT) based on P(PyV)‐H exhibited a gain value of 250, the highest reported among OECTs and organic field‐effect transistors (OFETs). The P(PyV)‐H‐based amplifiers recorded a clear cardiac cycle waveform during ECG measurement, with an SNR 40 times higher than that of commercial gel electrodes.^[^ ^76^ ^]^ The future of wearable technologies also requires biopotential monitoring electrodes that, if disposable, are environmentally friendly and do not contribute to electronic waste. In response to this need, Mirbakht et al. developed silk‐based ECG electrodes that biodegrade in soil within just two days.^[^ ^77^ ^]^ These electrodes also reduce skin irritation thanks to their high breathability and self‐adhesive properties.

In addition to material innovations, researchers have explored various structural designs, including electrode patterns and patch layouts, to further enhance device performance. Bioinspired electrode patterns have been introduced to improve skin adhesion and shape adaptability, drawing inspiration from natural systems such as fractal geometries,^[^ ^78^ ^]^ diving beetle architecture,^[^ ^79^ ^]^ gecko‐inspired hierarchical structure,^[^ ^80^ ^]^ octopus suction cups,^[^ ^81^ ^]^ and tree frog toe pads.^[^ ^82^ ^]^ These strategies enable accurate ECG signal acquisition under motion and in different environments, even underwater. Another critical factor in patch design is achieving high air and moisture permeability at the skin–device interface, which significantly influences user comfort and signal stability during prolonged use. Zhang et al. developed a new 3D liquid diode (LD), comprising an asymmetrically wetted micropillar array that enables unidirectional sweat transport, from the skin to the external environment, while preventing backflow.^[^ ^83^ ^]^ The proposed system comprised horizontal LD layers on top of vertical LD layers, mitigating sweat accumulation and facilitating superior unidirectional permeability. In a 3‐day wearability test, conventional ECG and polydimethylsiloxane (PDMS) patches caused visible erythema, whereas the 3D LD patch showed no irritation or inflammation. ECG signals captured by the 3D LD patch remained stable before and after 20 min of exercise, contrasting with SNR deterioration observed in other patch types. Most ECG patches feature 2D architectures, with components and interconnections arranged in a planar layout. To overcome the performance limitations of traditional 2D configurations, researchers have subsequently proposed 3D architectures. Jang et al. developed a 3D interconnection network based on helical microcoil structures, which provided a foundation for stretchable patches with low modulus, compact geometries, and enhanced functionality. It employed wireless power delivery via receiver antenna and supercapacitor storage to supply regulated energy. For communication, it utilized analog filters and amplifiers to preprocess sensor signals before Bluetooth transmission to a smartphone, showing an early integration of edge processing to reduce data load and enable continuous monitoring. However, the component layout was still confined to a single layer.^[^ ^84^ ^]^ Figure 2b illustrates the first ECG wearable sensor with true 3D integration proposed by Huang et al. in 2018, where both component distribution and interconnections were 3D.^[^ ^85^ ^]^ This system employed a four‐layer stretchable configuration fabricated via transfer printing of circuits onto elastomers. Vertical interconnect access (VIA) was realized through laser ablation, allowing components to be distributed in 3D. Its multilayered structure enabled higher integration density and more sophisticated levels of functionality of wearable ECG systems.^[^ ^85^ ^]^

E‐tattoo ECG systems can be seen as an advanced evolution of traditional ECG patches, distinguished by their lightweight construction, high conformability to the skin, and excellent breathability, which collectively contribute to the comfortable and imperceptible operation. Typically, the sensor component of an e‐tattoo is layered on a polymer substrate like polyethylene terephthalate (PET) coupled with a sacrificial layer such as polyvinyl alcohol (PVA).^[^ ^86^ ^]^ Initially, the polymer substrate provides structural support. It is then removed by dissolving the sacrificial layer in water, leaving the sensor attached to the skin via van der Waals forces. This intimate skin–device interface enhances SNR and tolerance to motion artifacts.^[^ ^87^, ^88^ ^]^ Kim et al. pioneered the first ECG e‐tattoo in 2011, integrating it with coils for wireless power and radio communication.^[^ ^87^ ^]^ Subsequent research has focused on advancing electrode materials (e.g., graphene,^[^ ^89^ ^]^ carbon nanotubes,^[^ ^90^ ^]^ Au,^[^ ^91^, ^92^ ^]^ and metal nanowires^[^ ^93^ ^]^), microstructures,^[^ ^86^ ^]^ and sensor configurations,^[^ ^94^ ^]^ to improve impedance, SNR, stretchability, and durability. After more than a decade of development, e‐tattoo systems have evolved from simple electrode pairs into fully integrated multifunctional platforms. For instance, Huttunen et al. have developed an ECG e‐tattoo with a dual‐pathway signal system, featuring an electronic circuit layout atop a 150 µm thermoplastic polyurethane (TPU) substrate, with the connection pad on the underside. A 75 µm PVA layer was used as a sacrificial layer for attaching the e‐tattoo to the skin. Key components such as a thin coin cell battery, microcontroller, analog front‐end, and Bluetooth radio were integrated into the TPU substrate, enabling wireless operation for up to 8 days.^[^ ^94^ ^]^ The multifunctionality of e‐tattoos has attracted considerable attention. Gogurla et al. developed an ultrathin multifunctional e‐tattoo that integrated sensing and stimulation capabilities, including temperature detection, heat generation, and ECG monitoring. The device was constructed on a 2 µm‐thick porous silk nanofiber network serving as a substrate, onto which single‐wall carbon nanotube (CNT) electrodes were deposited. This architecture enabled robust mechanical adaptability, maintaining functional stability under skin elongation and folding, while ensuring strong conformal adhesion. Leveraging both the Joule heating effect and high broadband optical absorption properties of CNTs, the device supported dual‐mode thermal stimulation via electrical and optical means. Notably, the CNT/SNF electrodes delivered a clear cardiac cycle waveform comparable to those recorded with commercial gel electrodes.^[^ ^90^ ^]^ Another growing trend is integrating ECG sensors with multimodal sensors to support multiparameter physiological monitoring.^[^ ^84^, ^85^, ^95^, ^96^ ^]^ For instance, a flexible skin patch has been developed to monitor ECG, temperature, hydration, blood oxygen, and pH levels simultaneously.^[^ ^96^ ^]^ Ha et al. developed a soft electro‐mechano‐acoustic cardiovascular sensing e‐tattoo capable of simultaneous ECG and seismocardiography (SCG) measurements. The device integrated serpentine Au electrodes and polyvinylidene fluoride (PVDF) vibration sensors for comprehensive cardiac monitoring. As shown in Figure 2c, the e‐tattoo maintained full functionality without delamination under repeated deformation, highlighting its excellent skin conformability.^[^ ^91^ ^]^

#### 3.1.2 Smart Textile Electrocardiography (ECG)

Textile‐based ECG systems utilize nylon, polyester, and cotton fabrics functionalized with electroconductive materials to serve as electrodes.^[^ ^72^, ^97^ ^]^ Unlike smart patches and e‐tattoos, which prioritize conformability and stable skin contact, textile‐based ECG systems offer advantages in convenience, comfort, and reusability, making them especially promising for long‐term ECG monitoring.^[^ ^98^, ^99^ ^]^ Textile‐based ECG signal acquisition relies on close contact between fabric electrodes and the skin, typically achieved by tight‐fitting garments that provide stable electrode–skin coupling. To ensure high signal quality and user comfort during long‐term wear, ECG e‐textiles have evolved from early designs using metal pads integrated through embroidering, sewing, and knitting, to more advanced materials and fabrication strategies.^[^ ^100^, ^101^, ^102^ ^]^ Researchers now employ soft, conductive materials such as conductive polymers, graphene and its derivatives, and carbon nanotubes, which offer excellent electrical performance while maintaining fabric flexibility and breathability.^[^ ^103^, ^104^, ^105^ ^]^ These materials are integrated into textile substrates using scalable and fabric‐friendly fabrication techniques, including dip coating, dyeing, screen printing, and spray painting with stencils.^[^ ^101^ ^]^ Such methods enable uniform material deposition while preserving the breathability and stretchability of the fabric.^[^ ^106^ ^]^ Additionally, encapsulating layers or stretchable binders are often applied to enhance mechanical durability and washability without compromising comfort or signal stability during extended use.

Liang et al. introduced a stable and biocompatible sericin‐CNT hybrid ink mediated by silk protein. This hybrid ink supports multiple electrode fabrication methods, including direct writing, stencil printing, and dyeing. This hybrid ink successfully enabled clear ECG signal acquisition on textile substrates.^[^ ^105^ ^]^ Beyond materials development, multifunctional e‐textiles have been designed to simultaneously monitor ECG and additional biosignals. Sharifuzzaman et al. developed a multimodal electrochemical–electrophysiological biosensing patch integrated into a textile platform. This system measured sweat glucose with pH adjustment while delivering high‐quality ECG signals (Figure 2d). A 3D fibrous carbon nanofiber network enriched with porous TiO_2_ particles was fabricated through laser carbonaceous thermal oxidation, serving as the ECG electrode. This composite exhibited a sheet resistance of 15.6 Ω sq^−1^ and delivered ECG signals with a high SNR of 37.63 dB.^[^ ^107^ ^]^

In addition to passive electrodes, fiber‐based active devices have also drawn increasing research attention. To address the low drain current output of conventional fiber‐shaped thin‐film transistors (TFTs), Kim et al. have proposed fibrous transistors with a double‐stranded assembly of electrode microfibers for textile ECG. Au microfiber, Poly(3‐hexylthiophene‐2,5‐diyl) (P3HT) coating, and Poly(vinylidene fluoride‐co‐hexafluoropropylene) (PVDF‐HFP) functioned as electrode, semiconductor layer, and gate dielectric, respectively. The double‐stranded configuration led to increased channel width and enhanced electrical contact between the semiconductor and the twisted electrodes. These transistors achieved a high current output of −5 mA under a −1.5 V operating voltage and demonstrated robust wash resistance, retaining sensing performance after one hour of detergent immersion at 550 rpm.^[^ ^108^ ^]^

#### 3.1.3 Intracardiac Electrocardiography (ECG)

In vivo measurements performed directly on the epicardium are of particular interest for recording ECG signals with high spatiotemporal resolution, potentially down to the level of individual action potentials.^[^ ^72^ ^]^ Such high‐resolution recordings enable precise localization of pathophysiological origins and can provide critical guidance during surgical interventions.^[^ ^109^, ^110^, ^111^ ^]^ **Table** **1** summarizes the structures and key performance metrics of representative intracardiac ECG (iECG) sensing devices.

**Table 1:** Summary of structural features and key performance metrics of recent intracardiac Electrocardiography (iECG) sensors.

Sensing Element	Signal Channel	Transistor Type or Electrode Geometry	Materials	Electric Property	Mechanical Property	Noise	Refs.
Transistor	5 × 5	Rubbery TFT	AgNWs/PDMS, P3HT‐NFs/PDMS and ion gel	Voltage gain: 0.968 On/off ratio: 5.247 × 10^3^	Young's moduli of AgNWs/ PDMS: 6.60 Mpa	60‐Hz notch filter was applied	[135]
Transistor	4 × 4	OECT	PEDOT:PSS and Au	Transconductance: 1.1 mS	Mechanically stable	SNR of 52 dB	[134]
Transistor	5 × 5	OECT and OFET	DNTT; AlxO; Al; Au; and PEDOT:PSS	Transconductance: 1.6 mS	NA	1 µA spikes with a noise of 50 nA	[136]
Transistor	18 × 22	NMOS	Si	On/off ratio: 10^7^ Voltage gain: 0.97	For a 5 mm bending radius, the strain is 0.0243%	Mean noise of 55 µV and an SNR over 42 dB	[132]
Transistor	8 × 8	OTFT	DNTT; self‐assembled monolayer; Au and gel/CNT	Amplifier gain: 400	Young's modulus of gel/CNT is ≈10 KPa	SNR is 64 dB after amplification	[137]
Passive electrode	8 or 32	Circular	Ag/Au/PEDOT:PSS/TPU nanocomposites	Conductivity: 5 × 10^5^ Ω/cm. Charge storage capacity: 32.8 mC/cm^2^	Intrinsically soft	Initial SNR is 70 ± 3 dB	[128]
Passive electrode	4	Serpentine	Pt	NA	Flexible	A Bandpass filter is applied	[120]
Passive electrode	6	Circular	Ag‐Au nanocomposite	Conductivity: 72600 S cm^−1^	Maximum of 840%	NA	[121]
Passive electrode	4 or 6	Bipolar recording	Poroelastic silicone composites	Conductivity: 7.72 ± 1.52 Ω∙sq^−1^	Mechanical modulus = 0.15 Mpa, bending stiffness <8.0 × 10^7^ GPa µm^4^	Bipolar recording configuration	[111]

iECG, intracardiac electrocardiography; OTFT, organic thin film transistor; OECT, organic electrochemical transistor; OFET, organic field‐effect transistor; NMOS, N‐type metal–oxide–semiconductor; AgNW, silver nanowire; PDMS, Polydimethylsiloxane; P3HT‐NFs, poly(3‐hexylthiophene‐2,5‐diyl) nanofibrils; PEDOT:PSS, poly(3,4‐ethylenedioxythiophene) polystyrene sulfonate; DNNT, dinaphtho[2,3‐b:2′,3′‐f]thieno [3,2‐b]thiophene. CNT, carbon nanotube; TPU, thermoplastic polyurethane; SNR, signal‐to‐noise ratio; Vds, drain‐source voltage; R/R_0_, resistance change; NA, not available.

Mechanical property mismatch and inadequate biocompatibility can trigger immune responses,^[^ ^112^, ^113^ ^]^ necessitating careful structure design, material selection, and fabrication strategies.^[^ ^114^ ^]^ To mitigate these issues, researchers have developed diverse implant architectures—such as sheets, meshes, balloons, and petal‐like designs—that better conform to the heart's mechanical properties.^[^ ^115^, ^116^, ^117^, ^118^, ^119^, ^120^ ^]^ Kim et al. proposed sponge‐like poroelastic ECG electrodes adaptable for custom direct ink writing. The sponge‐like silicone composite electrode was comprised of foam silicone resin embedded with Ag flakes plated with Cu, minimizing the risk of separation or delamination. The sponge structure from direct ink writing exhibited poroelastic behavior and an ultralow mechanical modulus (E < 30 kPa). Employing a bipolar recording configuration effectively suppressed common‐mode noise. A 6‐channel ECG sensor array enabled spatiotemporal mapping of epicardial iECG signals in murine in vivo experiments. The device showed good in‐vitro biocompatibility, with H9C2 cell viability comparable to controls, while removal of the Au overcoat reduced viability below 70%. 14‐day in‐vivo implantation induced chronic inflammation, with epicardial thickening increasing from 44.4 ± 8.3 µm to 645.9 ± 5.3 µm, which needs to be improved in future studies.^[^ ^111^ ^]^ Besides, researchers developed new materials for iECG implants to achieve better recording performances.^[^ ^111^, ^121^, ^122^, ^123^ ^]^ For example, Choi et al. developed a novel electrode material for iECG sensors by embedding gold‐coated silver nanowires in an elastomeric block‐copolymer matrix. This approach mitigated the adverse health effects and corrosivity of Ag electrodes in human tissue. The Au‐Ag nanowire composite showed excellent biocompatibility, with >90% cell viability in MTT assays across H9C2, CCD‐986sk, and L929 cell lines. After 21 days of subcutaneous implantation in rats, histological analysis revealed reduced fibrosis and immune cell infiltration versus the Ag‐only device. Besides enhancing biocompatibility, this approach also achieved an improved conductivity of 72600 S cm^−1^ due to the high aspect ratio of the nanowire and percolation network. Nanocomposite maintained stable conductivity in both air and physiological solutions over 3 weeks, highlighting the superior long‐term electrical stability. The designed ECG mesh sensor with six pairs of nanowire electrodes showed no performance degradation after a 30% cyclic stretching test, demonstrating excellent patch stability. In vivo tests on swine hearts confirmed their ECG monitoring and electrical stimulation capability.^[^ ^121^ ^]^ Wet physiological environments lead to delamination issues for stable and long‐term operation of implanted bioelectronics. Researchers have explored various fabrication methods to address this problem.^[^ ^124^, ^125^, ^126^ ^]^ Recently, Won et al. employed laser beam scanning to functionalize Poly(3,4‐ethylenedioxythiophene) sulfonate (PEDOT: PSS) electrodes on various polymer substrates. The absorbed laser at the interface led to phase separation in PEDOT: PSS and nanoscale interlocking between PEDOT: PSS and substrate. They achieved high wet conductivity (101.4 S cm^−1^) and wet adhesion forces (peel strength of 64.4 N m^−1^). Besides, the electrode patterning resolution of 5 µm allowed for application in microelectrode arrays. The ECG recordings from a 6‐electrode array on an *ex vivo* rat heart exhibited an SNR above 30. The epicardial microelectrode array maintained stable signal quality (less than 10% decrease) and electrochemical impedance after cleaning with 30 s ultrasonication, demonstrating its robustness and potential for reuse.^[^ ^127^ ^]^

Furthermore, integrating iECG sensing electrodes with cardiac stimulators (such as electric and optical stimulators) would allow for the simultaneous monitoring and control of cardiac function in a closed‐loop system.^[^ ^120^, ^122^, ^128^ ^]^ Ausra et al. proposed a wireless battery‐free implantable device with ECG recording, optogenetic light pacing, and computation capabilities. The device harvests wireless power via a 13.56 MHz primary antenna and stores energy in onboard capacitors to supply up to 33 mW peak load, just enough for multisite optogenetic stimulation and real‐time computation. It also features infrared‐based wireless communication to transmit processed heart‐rate data, enabling closed‐loop pacing and defibrillation in freely moving animals with millisecond precision. The device featured four petal‐like structures for multisite sensing and stimulation, each integrating one recording electrode (Pt) and three micro‐LEDs. All the other electronic components, including the primary antenna, capacitor bank, regulator, microcontroller, and analog front end, were interfaced on a flexible circular board. After enduring 100000 cycles of 5% strain, the petal arrays maintained their functionality, demonstrating their robustness and durability. The device exhibited favorable biocompatibility, as indicated by stable postoperative recovery with body weight returning to baseline and no major complications. Additionally, behavioral analysis via DeepLabCut revealed no significant changes in social interaction 12 days after implantation. Furthermore, in vivo experiments on freely behaving mouse models successfully recorded ECG signals. These signals were sent wirelessly to an IR receiver outside the mouse, opening new avenues for cardiac pathological studies and antiarrhythmic therapy.^[^ ^120^ ^]^ Sunwoo et al. developed a new implantable cardioverter‐defibrillator featuring a 32‐channel electrode array structure for ECG mapping and low‐energy subthreshold electrical stimulation (Figure 2e).^[^ ^128^ ^]^ Their system monitored ECG signals in patients at high risk of life‐threatening arrhythmias and administers painless ventricular tachyarrhythmia treatment. Before ventricular tachycardia treatment, capturing subthreshold ventricular tachycardia through ECG signal mapping is essential.^[^ ^129^ ^]^ ECG propagation delay and threshold voltages helped identify potential myocardial infarction sites. The 32‐channel array covered the entire ventricle, enabling detection of both anterior and posterior regions. Based on ECG mapping results, electrical stimulations were applied to terminate ventricular tachyarrhythmia. As shown in the inset of Figure 2e, the proposed electrode array was mounted on a rabbit heart, which recorded accurate ECG signals and provided effective ventricular tachycardia treatments. Simple suture fixation, as used in this study, may be insufficient for long‐term implantation, indicating the need for improved strategies such as hydrogels or mechanical fasteners, along with further validation of long‐term impedance stability and biocompatibility.^[^ ^128^ ^]^

With advances in iECG technology, researchers have increased electrode densities to acquire high‐resolution, high‐density signals; however, this increase may exacerbate crosstalk among power lines, thereby degrading signal quality.^[^ ^62^ ^]^ To address this, active transistor arrays are proposed as an alternative to passive electrodes. In these arrays, the ECG signal is captured at the gate of the transistors and amplified through the source‐drain current. Unlike passive electrodes, active transistor arrays require an additional power supply, further complicating the system. Still, the active transistor array enables simultaneous local ECG signal amplification and active matrix multiplexing, making them scalable.^[^ ^130^ ^]^ Fang et al. introduced a Si field effect transistor (Si FET) array, the first iECG array with multiplexing capability and capacitive coupling. This array integrated 396 multiplexed capacitive sensor elements. One sensing element consisted of two Si N‐type Metal–Oxide–Semiconductor (NMOS) transistors responsible for capturing ECG signals from tissue and selecting the captured signals in a time sequence. Figure 2f illustrates the capacitive‐coupled transistor design. An extra 900 nm thermal SiO_2_ served as the dielectric layer for capacitive coupling, significantly enhancing capacitance by more than one‐fold. This capacitive layer reduced the leakage from the transistor to below 10 ^−9^ A cm^−2^.^[^ ^131^ ^]^ This value was stable for 120 days of continuous operation in saline solution and biofluids. The device demonstrates excellent long‐term mechanical stability, maintaining consistent performance after up to 10000 bending cycles at a 5 mm radius, with no performance degradation or thermal accumulation during operation. *Ex vivo* testing involved attaching the 18 × 22 Si FET array to a rabbit heart model (Figure 2f), yielding controllable ECG signal detection with SNR above 42 dB. Foreign‐body response remains a concern, with risks of inflammation and fibrotic encapsulation affecting signal quality; strategies like anti‐inflammatory agents or triazole‐modified hydrogels are suggested. Additionally, while the system is flexible, enhanced stretchability is needed to conform to full‐organ geometries.^[^ ^132^ ^]^ Besides rigid Si FET, researchers also applied inherently soft transistor technology in iECG monitoring to enhance flexibility, leading to better conformal contact and deformation alongside heart pumping. Lee et al. developed an ultrathin (2.6 µm) active multi‐electrode array consisting of 4 × 4 OECTs for ECG mapping (Figure 2g). PEDOT: PSS functioned as an active layer on top of 70‐nm Au electrode. The transistor array was integrated on a parylene honeycomb substrate for higher mechanical stability and structural stretchability.^[^ ^133^ ^]^ Under 1000 times 15% cyclic strain, the stretchable OECT showed only a 7% decrease in transconductance and drain current, demonstrating excellent mechanical durability. These OECT achieved higher transconductance than the Si FET. The *ex vivo* test in a rats’ heart, achieved an SNR of 52 dB and a high stability (SNR = 51 dB after 30 min of attachment).^[^ ^134^ ^]^ To further enhance the system complexity, Sim et al. developed an intrinsically rubbery multifunctional epicardial patch. It incorporated a 5 × 5 TFT array, a strain sensor, a temperature sensor, and a mechanoelectrical transducer on PDMS substrate. The system had a Young's modulus similar to the heart tissue, and could still operate under 30% stretching. Proposed rubbery transistor maintained stable performance for 46 days with no significant degradation in key parameters, including field‐effect mobility, on‐current, or off‐current. For the ECG detection, the proposed TFT array utilized electrodes located at the source of the transistors. The TFTs array achieved active matrix multiplexing through transistor gate control (on/off). The epicardial patch incorporates a rubbery mechanoelectrical transducer that harvests energy from heartbeats. In‐vivo tests on a porcine heart demonstrated an open‐circuit voltage of 0.74 ± 0.01 V during pacing, while the short‐circuit current went up to 13.39 ± 2.59 nA, which could support long‐term multiplexed sensing and pacing. Figure 2h shows the sensing patch attached to an in vivo living porcine heart to validate the capability of multiplexed ECG mapping. In this study, the 5 × 5 ECG mapped the conduction velocities of heart electrophysiological signals.^[^ ^135^ ^]^

This section outlines the mechanisms and recent advancements in various ECG sensing formats, including wearable patches, e‐tattoos, textiles, and intracardiac implants. Researchers have increasingly focused on flexible ECG electrodes owing to their superior comfort, stretchability, and adhesion compared to conventional rigid metal electrodes, such as pre‐gelled Ag/AgCl. The primary research focuses include material modifications (e.g., tissue‐electronics interfaces and semiconducting polymers) and structural designs (e.g., 3D low‐dimensional configurations and 3D integration). These approaches synergistically enhance the performance and functionality of ECG patches. E‐tattoos offer improved permeability and conformability over conventional ECG patches, leading to higher SNR and reduced discomfort. However, their durability remains challenging, as the seamless contact limits sweat evaporation, potentially compromising long‐term stability. For high‐precision ECG signal acquisition, implantable devices enable direct epicardial mapping. A key challenge in this field is the mechanical, thermal, and chemical mismatch between iECG implants and heart tissue. To address this, researchers have explored various configurations (e.g., 2D sheets, balloons, petals, sleeves, and meshes) and materials (e.g., nanowires, nanocomposites, and porous electrodes) to improve conformal contact with the epicardial surface, enabling real‐time spatiotemporal mapping. Additionally, replacing passive electrodes with active transistor arrays has been proposed to achieve high‐resolution, high‐density ECG signals while minimizing crosstalk among power lines. Beyond integrating iECG sensing with pacemakers and defibrillators for simultaneous monitoring and therapy, researchers are developing multifunctional implantable systems capable of monitoring additional parameters (e.g., temperature and strain) and facilitating treatments (e.g., ablation). Additionally, combining tissue engineering with iECG implants offers a promising avenue for real‐time monitoring and stimulation‐driven regeneration of damaged myocardial tissue.

### 3.2 Biopotentials of Skeletal Muscles: Electromyography (EMG)

Electromyography (EMG) records the cumulative action potentials generated by motor neurons during muscle fiber activation. EMG serves as a critical tool for diagnosing musculoskeletal disorders and neurodegenerative diseases, as well as for monitoring rehabilitation progress, physiological training, and prosthetic control.^[^ ^28^, ^138^, ^139^ ^]^ EMG techniques are broadly categorized into surface EMG (sEMG) and intramuscular EMG (iEMG). sEMG is a non‐invasive method that uses electrodes placed on the skin surface, typically capturing signal amplitudes ranging from 0.01 to 10 mV.^[^ ^28^ ^]^ In contrast, iEMG uses needle‐type or thin‐film electrodes to acquire more localized EMG signals with higher SNR, enabling the detection of activity from individual muscle fibers or motor units.

#### 3.2.1 Surface Electromyography (EMG)

Various structures, including bipolar electrodes, multichannel electrode arrays, and active sensing nodes such as transistors, capture sEMG signals through the skin‐electrode interface. sEMG wearables are categorized into three primary types: smart patches, e‐tattoos, and e‐textiles.^[^ ^62^ ^]^ Sharing similar specifications with ECG wearables, these sensors have driven significant research into material properties and microstructures to enhance sensor performance. This has led to extensive investigations into various aspects of wearable electrophysiological sensors for both ECG and EMG applications.^[^ ^84^, ^85^, ^86^, ^87^, ^89^, ^90^, ^140^, ^141^, ^142^, ^143^, ^144^, ^145^ ^]^ Compared to ECG, sEMG electrodes face three distinct challenges that necessitate different material and structural design strategies. First, sEMG signals exhibit frequencies up to 500 Hz and lower amplitudes in the range of 0–1.5 mV. Therefore, sEMG electrodes require materials with higher conductivity and more careful interface engineering to minimize parasitic capacitance.^[^ ^146^ ^]^ Second, unlike ECG, sEMG sensors demand higher spatial resolution to resolve motor unit potentials, driving the need for high‐density microelectrode arrays. Third, sEMG electrodes must withstand greater mechanical deformation because of muscle contractions. This is achieved by using soft, stretchable substrates with mechanical decoupling architectures (e.g., serpentine interconnects). Therefore, EMG systems, particularly sEMG, must address issues such as crosstalk from adjacent muscles and variability in skin surface topography, both of which can distort signal fidelity and complicate interpretation. **Table** **2** summarizes the structural features and key performance metrics of representative wearable EMG electrodes.

**Table 2:** Summary of structural features and key performance metrics of wearable Electromyography (EMG) sensors.

Category	Signal Channel	Electrode Material	Electrode Geometry	Electric Property	Stretchability	Noise	Refs.
Patch	8	CNT/silicone	Square	Electrical impedance: 150 Ω	Maximum 50%	Signal amplitude is −55 dBm at 0.5 m	[84]
Patch	8	Supramolecular additive; PEDOT:PSS	Square	Conductivity: 2700 S cm^−1^ (no strain) and 6000 S cm^−1^ (at 100% strain)	600%	High SNR and low signal variance	[148]
Patch	21	Au nanoparticle on SEBS	Circular	Sheet resistance < 10 Ω sq^−1^	600%.	SNR: during pressing (17.2 dB)	[147]
E‐tattoo	16	PET metalized by Cr and Au	Square	NA	Maximum 560%	Background noise is 5 µV	[142]
E‐tattoo	2	PEDOT:PSS	Circular	Sheet resistance<520 Ω sq^−1^	The first failure is at 8%.	SNR is 6.45 dB	[143]
E‐tattoo	1	CVD graphene on PMMA	Circular	Sheet resistance: 1994.33 ± 264 Ω	Up to 50%	SNR is 15.22 dB	[89]
E‐textile	1	PVDF nanofibers, fluoroelastomer, and silver flakes	Transmission line	Conductivity: 9903 S cm^−1^ Sheet resistance: 0.047 Ω.sq^−1^	More than 300%	The initial noise level is 75 µV (peak‐to‐peak)	[163]
E‐textile	4	Silver‐fluoroelastomer/tricot knit textile	Circular	Sheet resistance: 0.06 Ω.sq^−1^	Up to 450%	The noise level is 0.07 mV under compression	[164]
E‐textile	1	Ag/Au on PET/cotton fabric	Square	100‐Hz impedance is 70 Ω	NA	The initial SNR is 12.5 dB	[165]

EMG, electromyography; PDMS, Polydimethylsiloxane; SEBS, styrene and ethylene/butylene; CNT, carbon nanotube; PVA, polyvinyl alcohol; HPPNF, hierarchical polyaniline/polyvinylidene fluoride nanofiber; PEG‐Dopa, dopamine‐functionalized poly(ethylene glycol); PVDF, Polyvinylidene; PET, Polyethylene terephthalate; PLLA, Poly‐L‐lactide; PLA, polylactic acid; TFT, thin film transistor; FBG, fiber Bragg grating; GF, gauge factor; R2, linearity; NA, not available.

**Figure 3:**
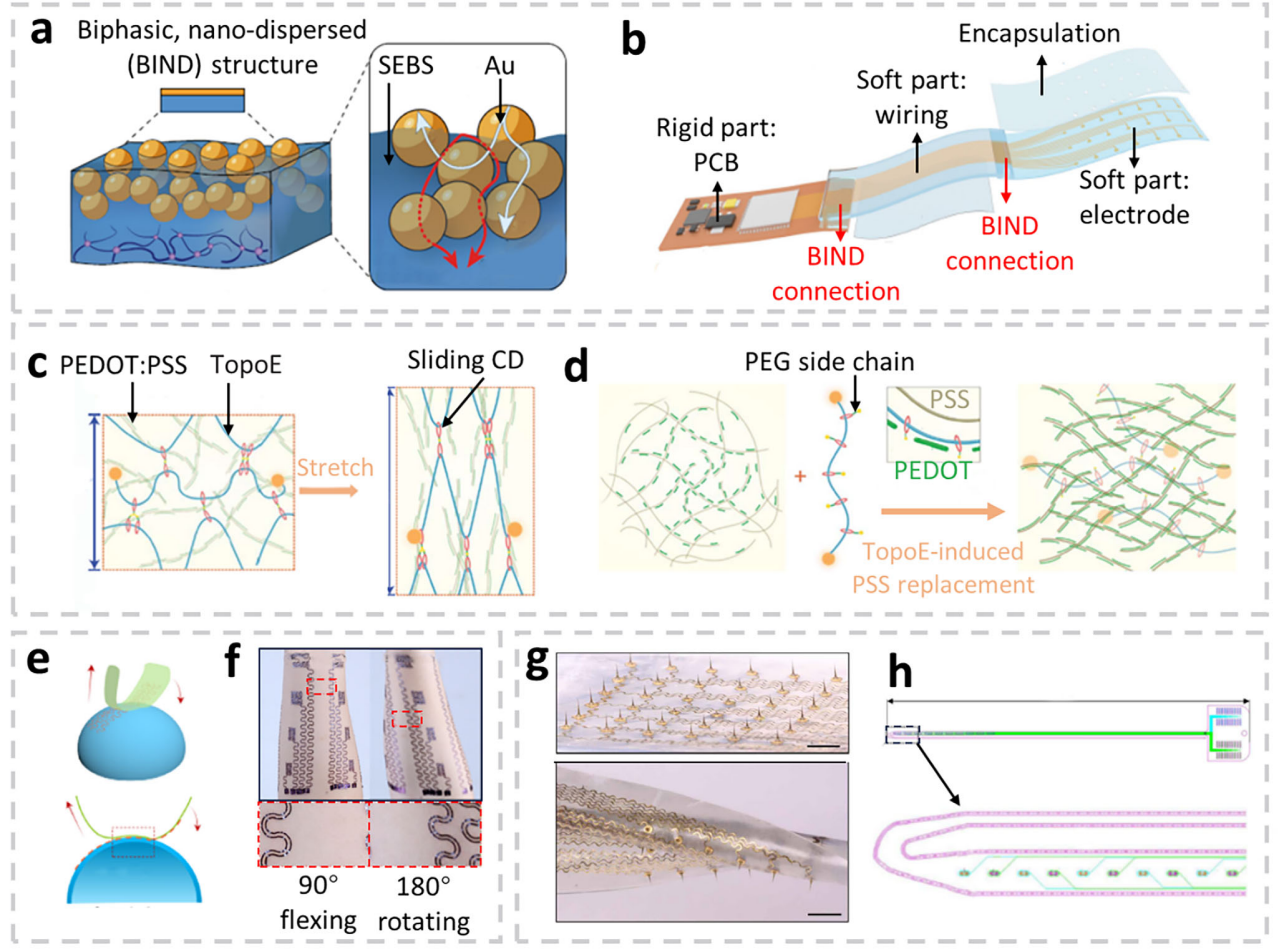
Wearable and implantable sensors dedicated to Electromyography (EMG) signal measurement. a) The illustration of the biphasic, nano‐dispersed (BIND) interface composed of Au and styrene‐ethylene‐butylene‐styrene (SEBS), where red and white arrows represent the continuous mechanical and electrical pathways.^[^ ^147^ ^]^ b) Schematic of 21‐channel EMG electrode array where the rigid part (PCB), soft part (wiring and electrodes), and encapsulation part are connected through BIND interface.^[^ ^147^ ^]^ c) Schematic illustrating how TopoE increases the stretchability of Poly(3,4‐ethylenedioxythiophene) sulfonate (PEDOT:PSS) through sliding cyclodextrins (CDs).^[^ ^148^ ^]^ d) Schematic diagram of PEDOT: PSS conductivity enhancement thanks to TopoE‐induced PSS replacement.^[^ ^148^ ^]^ e) Cartan transfer printing (CTP) of e‐tattoo pattern on a 3D surface.^[^ ^142^ ^]^ f) Photographs of e‐tattoo attached to 90° flexing and 180° rotating forearm through CTP. The inset shows the local magnification of the e‐tattoo pattern on the skin.^[^ ^142^ ^]^ g) Optical image of the stretchable micro‐needle electrode array (MNEA) on silicone substrate without strain (top) and under stretching and twisting (bottom). Scale bar: 3 mm.^[^ ^155^ ^]^ (h) A high‐density intramuscular EMG array with 40 platinum electrodes was manufactured on both sides of the probe.^[^ ^156^ ^]^ All pictures are reproduced with permission.

Advances in material science play a pivotal role in driving the development of sEMG electrodes.^[^ ^147^, ^148^, ^149^, ^150^, ^151^, ^152^ ^]^ Jiang et al. introduced a biphasic, nano‐dispersed (BIND) interface by thermally evaporating Au nanoparticles within self‐adhesive styrene‐ethylene‐butylene‐styrene (SEBS, **Figure** **3**a). Owing to its unique interfacial material composition, BIND successfully integrates rigid, soft, and encapsulating components within wearable EMG sensors. This design ensures strong interface adhesion, maintaining reliable connections under strains exceeding 600%, and offers low electrical resistance (less than 4 Ω at 50% strain). The researchers fabricated a 21‐channel EMG device comprising soft electrodes, a rigid polyimide printed circuit board (PCB), and encapsulation components, integrated using a BIND interpenetrating structure (Figure 3b). The assembled sensor achieved EMG detection with an SNR of 20.9 dB and mechanical resilience (consistent SNR pre‐ and post‐pressure application). This innovative interpenetrating structure simplifies the design process and enhances the design versatility of stretchable devices.^[^ ^147^ ^]^ Researchers pursue high mechanical robustness and good electrical conduction in organic EMG electrodes. Combining these two properties in a single material remained challenging until Jiang et al. proposed a topological supramolecular network to decouple the competing effects. They designed a special polyrotaxane additive called TopoE to modify the property of PEDOT: PSS. TopoE consists of a polyethylene glycol (PEG) backbone with PEG methacrylate side chains functionalized with sliding cyclodextrins (CDs), forming a stretchable network shown in Figure 3c. This configuration significantly enhanced the stretchability of PEDOT: PSS/TopoE composites to 150%. Figure 3d illustrates how TopoE enhances conductivity by replacing part of the PSS with the polar PEG side chains, leading to increased PEDOT aggregation. The conductivity of PEDOT: PSS/TopoE increased by two orders of magnitude compared to that of pristine PEDOT: PSS. Increasing TopoE content resulted in higher stretchability, lower Young's moduli, and better conductivity, which were favorable for wearable EMG electrode applications. Based on this novel polymer design, Jiang et al also developed an 8‐channel sEMG array with a small interelectrode distance (500 µm), achieving consistent high‐fidelity EMG signal recording with high spatial resolution.^[^ ^148^ ^]^

Mathematical concepts have also contributed to the advancement of EMG wearables. Wang et al. extended a 2D mathematical concept, Cartan development, to the 3D curvilinear skin surface. This innovative approach led to Cartan transfer printing (CTP), which facilitated the conformal contact of large‐area e‐tattoos. Figure 3e illustrates how CTP involved gradually rolling a donor substrate along the e‐tattoo sensor to achieve conformality while maintaining the fixed 3D surface. A large‐area EMG e‐tattoo with a thickness of 1.2 µm was adhered seamlessly to the arm. CTP ensured no skin wrinkling even when flexed at angles of 90° and 180° (Figure 3f). The EMG signals measured with this device exhibited background noise levels below 5 µV.^[^ ^142^ ^]^

#### 3.2.2 Intramuscular Electromyography (EMG)

iEMG technology offers significantly higher spatial resolution, enabling the detection of action potentials at the level of single motor units located deep beneath the skin surface.^[^ ^153^ ^]^ Despite its limited accessibility due to safety concerns and operational complexity, iEMG remains widely used in clinical settings. Invasive needle and probe‐like wired electrodes are the two primary types used in clinical settings.^[^ ^28^ ^]^ Recent advances in microfabrication technologies have catalyzed the development of novel micro‐needle electrode arrays (MNEAs) and implantable electrode array probes (EAPs) for iEMG sensing techniques, aiming at overcoming the inconvenience and limited coverage area.^[^ ^154^, ^155^, ^156^, ^157^, ^158^, ^159^, ^160^, ^161^, ^162^ ^]^

MNEAs provide a minimally invasive approach to recording iEMG signals by penetrating the stratum corneum (the outermost layer of the epidermis) and directly detecting signals within the epidermal layer. MNEAs effectively mitigate impedance at the skin‐electrode interface and enhance measurement stability during dynamic movement. However, most MNEAs are constructed on rigid substrates (e.g., silicon, ceramic, and glass), which limits their conformability to the skin.^[^ ^134^ ^]^ The challenge of integrating electrode fabrication processes with stretchable substrates has hindered the development of MNEA on soft substrates. The advancements in flexible substrate materials such as PDMS, parylene, polyimide, and photoresist (SU‐8) have addressed this mechanical mismatch issue.^[^ ^159^, ^161^, ^162^ ^]^ Recently, Zhao et al. developed a highly stretchable and customizable MNEA on a silicone substrate, achieving outstanding stretchability across the entire sensor. At the same time, it maintained a high Young's modulus in the microneedles. Covalent bonding between microneedles and silicone, together with a serpentine interconnection design, allowed up to 90% stretchability without delamination during stretching and twisting (Figure 3g). Besides, the controllable Cu dissolving through gel etchant led to customizable sensing areas of needle electrodes. The microneedles exhibited a high Young's modulus (E = 6.6 GPa), facilitating robust tissue insertion without buckling. Successful recording of iEMG from the buccal mass of Aplysia showed the potential for wearable iEMG and implantable applications.^[^ ^155^ ^]^ In contrast to MNEAs that are minimally invasive and attached to the skin or tissue, implantable EAPs are inserted deep into tissue areas to capture multichannel iEMG signals. Moreover, implantable EAP devices have the potential to detect single motor units, enabling precise mapping of neural drive and offering improved neurophysiological understanding, as well as enhanced accuracy in prosthetic control, motor disorder diagnosis, and human‐machine interface applications through detailed monitoring of individual motor unit activity. Muceli et al. developed an iEMG array, integrated 40‐channel thin‐film electrodes with 0.5 mm interelectrode spacing and microrough platinum coating on both sides of a polyimide substrate. Its high‐density electrode distribution significantly improved spatial resolution and localization, enabling the concurrent detection of up to 45–50 motor units per array (Figure 3h). These arrays can sample from both deep and superficial muscle regions, capturing action potentials across multiple channels, which increases decomposition accuracy. The probe offered a spatial resolution of 0.5 mm, enabling the recording of electrical activity from within the anatomical territory of individual motor units and allowing localization to each unit. Coupled with an automated decomposition algorithm, this high‐density multichannel iEMG EAP facilitated the study of spinal motoneuron behaviors in vivo.^[^ ^156^ ^]^

Significant progress has been made in EMG sensing, driven by applications in disease analysis, rehabilitation (e.g., neuromuscular disorders), and robotics control. Wearable EMG follows a development trajectory similar to that of wearable ECG, emphasizing improvements in sensing performance, convenience, and comfort. Researchers have explored novel materials (e.g., nano‐dispersed material interfaces^[^ ^147^ ^]^ and topological supramolecular networks^[^ ^148^ ^]^) and structural innovations (e.g., Cartan transfer printing^[^ ^142^ ^]^) to enhance adhesion, breathability, flexibility, and biocompatibility. Biomedical researchers are particularly interested in iEMG at the single‐cell level for a deeper understanding of physiological mechanisms. However, achieving stable long‐term in *vivo* signal recording at this scale remains challenging. Enhancing electrode‐cell interfaces is essential for improving iEMG sensing performance at the single‐cell level. Signal stability remains a critical challenge in both wearable and implantable EMG sensing. In wearable EMG, impedance variations, motion artifacts, surface topography, and crosstalk from neighboring skeletal muscles contribute to signal instability. Various approaches have been investigated to improve signal quality, including the use of more skin‐conformal electrodes, advanced signal processing (e.g., denoising and artifact subtraction), and spatial filtering techniques (e.g., double‐differential electrode configurations). In iEMG, instability arises from poor reproducibility due to small monitoring regions and foreign body responses. Machine learning techniques, particularly pattern recognition‐based algorithms, offer a promising approach to mitigating the challenge.

### 3.3 Biomagnetic Signals Monitoring: Magnetomyography (MMG)

In 1972, scientists defined magnetomyography (MMG) as the magnetic field vectors originating from the local currents associated with EMG signals in skeletal muscles.^[^ ^166^ ^]^ MMG and EMG are correlated by the Maxwell‐Ampere law. Compared to EMG, MMG technology has progressed rather slowly due to the low signal magnitude (pT‐fT) and complex measurement procedures.^[^ ^167^ ^]^ Nevertheless, the high spatial resolution of MMG and its capability for remote, contactless signal acquisition continue to attract research interest. MMG signals are increasingly being explored for applications in health monitoring, rehabilitation, robotic control, and diagnosis.^[^ ^168^ ^]^ Current MMG acquisition methods rely on highly sensitive devices such as superconducting quantum interference devices (SQUIDs) and optically pumped magnetometers (OPMs), which offer precise magnetic field detection but are costly, bulky, and require operation in shielded environments.^[^ ^166^, ^169^ ^]^ To address these limitations, recent efforts have focused on developing miniaturized MMG sensors based on thin‐film spintronic devices.^[^ ^170^, ^171^, ^172^ ^]^

The two most commonly employed mechanisms in spin‐based MMG sensors are magnetoelectric (ME) and magnetoresistive (MR). ME devices exploit mechanical resonance to convert magnetic fields into electric polarization, offering passive measurement capabilities and a wide linear range. This functionality is achieved through a combination of magnetostrictive and piezoelectric effects.^[^ ^173^ ^]^ To capture MMG signals across frequencies ranging from 10 to 300 Hz, the resonant frequencies of ME devices are adjusted through various device designs.^[^ ^170^, ^174^ ^]^ In contrast, MR devices change their electrical resistance in response to magnetic fields. MR sensors come in several types, including anisotropic MR (AMR), giant MR (GMR), and tunnel MR (TMR), each characterized by distinct magnetoresistance properties.^[^ ^175^ ^]^ Initially discovered in bulky metals such as Ni, AMR sensors exhibit low magnetoresistance levels of 2%–6%. GMR sensors are structured with FM/M/FM three‐layer configuration, where FM is a ferromagnetic material and M is a metallic material. GMR sensors show higher magnetoresistance values ranging from 6‐20%.^[^ ^175^ ^]^ The electrical resistance of GMR devices varies with the relative angle between the magnetization axes of the two ferromagnetic materials. Researchers have developed a GMR microprobe for local detection of the MMG signal generated by the action potential in the *ex vivo* mouse skeletal muscle. The measurements aligned well with theoretical predictions, validating the potential of GMR sensors for MMG signal capture.^[^ ^171^ ^]^ TMR sensors, which feature a similar 3‐layer structure but include an insulating barrier layer instead of a metallic spacer, achieve the highest levels of magnetoresistance (up to 300% with a MgO barrier).^[^ ^175^, ^176^ ^]^ Vertical electrical resistance is a function of relative magnetization orientations between the two ferromagnetic layers. Zuo et al. developed an integrated TMR sensor with pT resolution for MMG recording. This miniaturized sensor, comprising 58 × 19 elements, records MMG signals as low as 200 pT with an SNR exceeding 20. Alignment between experimental results and 3D finite element method simulations confirms the feasibility of this TMR sensor for MMG applications.^[^ ^172^ ^]^ The compatibility of thin‐film spintronic sensors with silicon‐integrated circuit technology suggests the potential for integrating MMG sensors with complementary metal‐oxide‐semiconductor (CMOS) readout circuitry. This integration would facilitate signal processing, amplification, and noise reduction.^[^ ^177^, ^178^ ^]^

Significant efforts have been made to optimize the physical and functional properties of MMG sensors through advancements in device design and material selection. A major challenge in MMG sensing is its relatively low SNR, primarily due to interference from geomagnetic fields (micro‐Tesla range) and environmental magnetic noise (tens of nano‐Tesla/√Hz). To mitigate this issue, researchers have employed shielding techniques (e.g., electromagnetic coils for noise cancellation), reference array channels (e.g., proportional‐integral‐derivative algorithms), and advanced signal processing methods. Skeletal muscles contractions and movements introduce additional noise, as changes in muscle dimensions alter the sensor‐to‐EMG current distance, impacting MMG signal stability. For implantable MMG sensors, human‐machine interfacing presents a more viable application, as prosthetic limb control involves less muscle movement, reducing motion‐induced signal disturbances.

## 4 Biomechanical Signals Monitoring

Biomechanical signals arise from the dynamic interplay of muscle contraction and relaxation, driven by action potentials. These signals reflect mechanical changes within muscle tissue and offer valuable insight into muscle physiology and function status.

### 4.1 Force Sensing: Force Myography (FMG)

Force Myography (FMG) detects variations in muscle volume during contraction and movement, providing detailed information about muscular activities.^[^ ^179^ ^]^ FMG has been widely applied in gesture recognition, prosthetic control, and human–machine interface systems.^[^ ^180^ ^]^ It employs both stress and strain sensors to capture force‐related data, with overlapping sensing mechanisms in many implementations.^[^ ^181^ ^]^ Common FMG sensing technologies include piezoresistive, piezoelectric, capacitive, and optical fiber‐based sensors. Significant research has focused on optimizing sensor layouts, materials, fabrication techniques, and data processing algorithms to enhance FMG performance.^[^ ^180^, ^182^ ^]^ This section reviews recent advancements in materials and structural design across the four primary force‐sensing modalities used in FMG. In addition, A summary of the structural features and key performance metrics of representative wearable and implantable FMG sensors is provided in **Table** **3**.

**Table 3:** Summary of structural features and key performance metrics of representative wearable and implantable Force Myography (FMG) sensors with four different sensing mechanisms.

Mechanism	Sensor Structure	Material	Sensitivity (S) and Gain Factor (GF)	Linear Response	Working Window	Stretchability	Refs.
Piezoresistive (stress)	Two‐layer thin film with topographic design	CNT; Ti_3_C_2_T_x_ MXene nanosheets; PVA	Maximum 3400 GF	12%–14% to 45–69%	6%–84%	50%	[197]
Piezoresistive (strain)	Three‐layer films	HPPNF; Au/PDMS	S of 53 kPa^−1^	Good linear response	58.4‐960 Pa	NA	[196]
Piezoresistive (strain)	Single‐layer thin film on flexible PCB	PEG‐Dopa; genipin‐crosslinked gelatin	GF: 1.02–1.46	R^2^ = 0.997–0.998	0‐100%	NA	[198]
Piezoelectric (strain)	6‐layer films with kirigami patterns	PVDF; Ag; PET	S: 9.86 V cm^−2^	Maximum R^2^: 0.9824	NA	320.80%	[205]
Piezoelectric (stress)	8 × 16 TFT array	ZnO; Cr/Au; polyimide	S: 0.0635 %/mN	Up to 250 mN	NA	NA	[207]
Piezoelectric (stress)	7‐layer film	PLLA; Mo; Mg; PLA	NA	Two linear regions	0‐18 kPa	NA	[239]
Capacitive (strain)	Three‐layer film	Adhesive elastomer; parylene/Au	GF: 3.05	R^2^ = 0.98	0%–140%	140%	[218]
Capacitive (stress)	Three‐layer film	PVDFHFP/[EMIM][TFSI]; Au/PDMS	S: 3103.5 kPa^−1^	1–34 kPa	NA	NA	[222]
Capacitive (strain)	Two fibers organized in the helical structure	Ag/polyurethane	GF: 12	Non‐linear	15‐27.5%	30%	[224]
Optical (strain)	5 × 5 strain sensing mesh using ten fibers	SEBS/carbon black particles	The highest GF was in 1960	NA	0%–400%	580%	[234]
Optical (strain)	Three‐layer laminate structure.	Silicone; SMF‐28Eþ type FBG	S: 27.42 pmN^−1^ for pressure. 139.3 pm mm^−1^ for strain	0–3.5N	Pressure: 0–3.5 N. Strain: 0‐20%	Up to 20%	[233]
Optical (stress and strain)	Core‐cladding structured fiber	Silicone; PDMS; ZnS: Cu phosphor	S: 0.0064	10% to 50% with R^2^ = 0.9938	10%–50%	85%	[235]

FMG, force myography; PDMS, Polydimethylsiloxane; SEBS, styrene, and ethylene/butylene; CNT, carbon nanotube; PVA, polyvinyl alcohol; HPPNF, hierarchical polyaniline/polyvinylidene fluoride nanofiber; PEG‐Dopa, dopamine‐functionalized poly(ethylene glycol); PCB, Printed circuit board; PVDF, Polyvinylidene; PET, Polyethylene terephthalate; PLLA, Poly‐L‐lactide; PLA, polylactic acid; TFT, thin film transistor; FBG, Fiber Bragg Grating; S, sensitivity; GF, gauge factor; R^2^, linearity; NA, not available.

#### 4.1.1 Force Sensing with Piezoresistive Sensors

Piezoresistive sensors function by converting mechanical forces into changes in electrical resistance through piezoresistive materials.^[^ ^183^ ^]^ These force‐induced resistance variations result from changes in material deformation, the number of conductive pathways, and contact resistance.^[^ ^184^, ^185^, ^186^ ^]^ Due to their inherent flexibility, piezoresistive sensors represent the predominant sensing mechanism used in FMG applications.^[^ ^182^ ^]^

**Figure 4:**
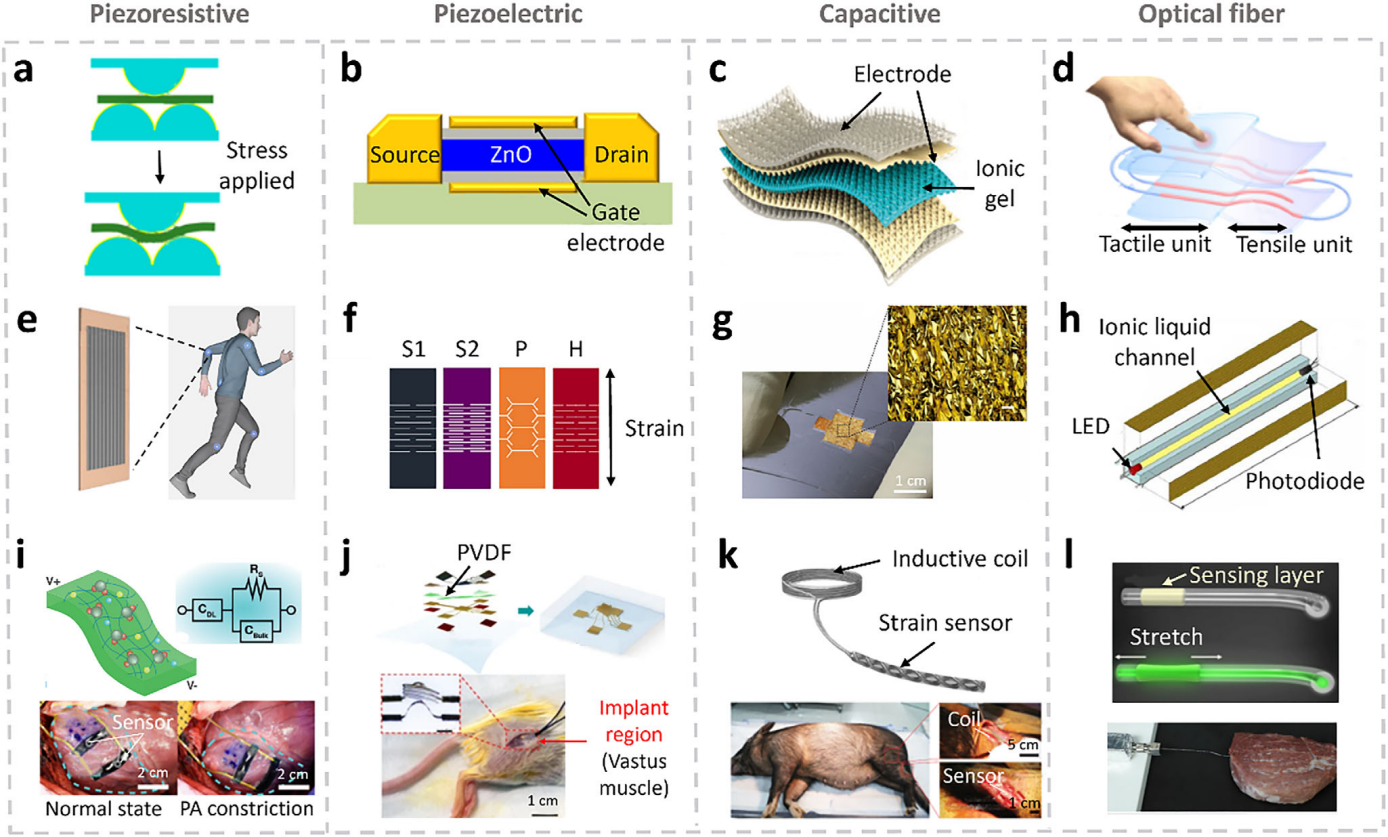
Wearable and implantable sensors for Force Myography (FMG) signal monitoring. a) Schematic illustration of the contact area change under stress, leading to resistance change.^[^ ^196^ ^]^ b) Schematic illustration of ZnO thin film transistor (TFT) force sensor.^[^ ^207^ ^]^ c) Schematic illustration of a multilayer capacitive stress sensor with a microcone pattern.^[^ ^222^ ^]^ d) Structure of the skin‐like optical fiber sensor with four fiber Bragg grating (FBGs) for both tensile and tactile sensing.^[^ ^233^ ^]^ e) Wrinkle‐like piezoresistive strain sensors attached to body joints for accurate full‐body avatar reconstruction.^[^ ^197^ ^]^ f) Schematic of four different Kirigami patterns used for a piezoelectric strain sensor.^[^ ^205^ ^]^ g) Optical image of strain sensor in the relaxed compressed state without applied strain. The inset shows the optical micrograph of a wrinkled Au thin‐film electrode at 0% strain.^[^ ^218^ ^]^ h) A multifunctional sensor with critical components for three heterogeneous sensing.^[^ ^230^ ^]^ i) Schematic of piezoresistive hydrogel strain sensor and corresponding Randles circuit model (top) and image of hydrogel strain sensors attached to the porcine ventricles in vivo under two different states (bottom).^[^ ^198^ ^]^ j) The exploded 2D view and 3D schematic illustration of the piezoelectric force sensor (top) and optical image of the 3D sensor implanted into the hind leg of the mouse in vivo (bottom).^[^ ^209^ ^]^ k) The wireless capacitive fiber sensor for strain sensing integrated with a coil (top) and a photograph of the pig used for in vivo sensing experiment. The inset shows the coil and sensor implanted in the posterior leg and thigh, respectively (bottom).^[^ ^224^ ^]^ l) Schematic illustration of stretchable mechanoluminescent optical fiber strain sensor in the static state and stretched state (top) and photograph of optical sensor implanted and tested in the *ex‐vivo* pork muscle tissue.^[^ ^235^ ^]^ All pictures are reproduced with permission.

Common piezoresistive materials include conductive polymers, fabrics, foam, and ionic liquids.^[^ ^187^ ^]^ Researchers have employed various microstructures such as pyramids,^[^ ^188^ ^]^ hemispheres,^[^ ^189^ ^]^ and hollow spheres^[^ ^190^ ^]^ for stress sensing, as well as misplacement,^[^ ^191^ ^]^ disconnection,^[^ ^192^ ^]^ and cracking^[^ ^193^ ^]^ for strain sensors to enhance sensing sensitivity. The naturally bioinspired microstructures also inspired the scientists to further improve the sensor performance.^[^ ^194^, ^195^ ^]^ For example, Yang et al. developed a flexible stress sensor referring to the microdome pattern of the rose petal. It featured a piezoresistive nanofiber film layer sandwiched between two electrode layers with a microdome structure. This sensor demonstrated high sensitivity (53 kPa^−1^) due to its unique microstructural design, where the contact tunnelling resistance between the nanofiber layer and microdome electrode varied with applied stress. **Figure** **4**a illustrates the increase in contact area in this system under stress, leading to decreased resistance. They showed that these sensors successfully captured FMG signals when applied to the biceps.^[^ ^196^ ^]^ During full‐body motion, the muscle deformation values differ at various joints/muscles. Therefore, the working window of the sensors needs to be customized in order to match the strain change at specific joints/muscles. To achieve full‐body muscle motion monitoring, Yang et al. have developed a piezoresistive Ti_3_C_2_T_x_ MXene nanolayer with tunable wrinkle‐like topographies through localized thermal contraction. This topographical design allowed the strain sensors to operate within a wide working window (6% to 84%), accommodating the range of human joint deformations. The proposed sensor achieved a sensitivity with gauge factors exceeding 1000. By tuning wrinkle density and distribution, the device achieves crack‐controlled piezoresistive response, offering both high linearity (R^2^ > 0.995) and durability over 10000 cycles. When integrated with on‐chip machine learning, these sensors formed an edge sensor module capable of real‐time in‐sensor avatar animation reconstruction. Figure 4e demonstrates these sensors as attached to various joints to monitor localized FMG signals. The reliance on thermal contraction to form MXene wrinkles poses challenges in achieving precise and reproducible large‐scale pattern uniformity across batches.^[^ ^197^ ^]^

Researchers have explored implantable piezoresistive sensors to detect localized muscle deformation with higher SNR. Song et al. proposed an implantable strain sensor based on a conductive hydrogel for monitoring myocardial contractions. The hydrogel, containing water as the mobile phase, was modeled using the Randles circuit (Figure 4i), which included components such as double‐layer capacitance (C_DL_), electrolyte solution resistance (R_s_), and bulk capacitance (C_bulk_). The piezoresistive hydrogel sensor operated at a high sampling rate of 10 kHz, exhibiting nearly pure resistive impedance with no detectable hysteresis. Proposed ionically conductive hydrogels showed high stretchability (>500%) and low modulus (<100 kPa). Scanning laser confocal fluorescence microscopy revealed high viability of C2C12 myoblasts on hydrogel substrates, with 95.1 ± 7.4% for Gelapin hydrogel, comparable to the 93.7 ± 4.0% observed in the control group. For in vivo validation, the hydrogel sensor was attached to the epicardial surface of a porcine heart to record strain during myocardial contraction (Figure 4i). To induce controlled cardiac strain, the pulmonary artery was transiently compressed for 10 s. The sensor successfully captured strain signals associated with left ventricular contractions.^[^ ^198^ ^]^

#### 4.1.2 Force Sensing with Piezoelectric Sensors

Piezoelectricity finds extensive application in both sensing and transduction, where an applied force generates a potential difference across the piezoelectric material. This potential results from altered dipole separations within the intrinsic noncentrosymmetric structure of piezoelectric materials.^[^ ^199^ ^]^ Unlike piezoresistive and capacitive sensors, piezoelectric sensors operate without an external power supply but rely on dynamic mechanical stimulation to generate a potential.

Common piezoelectric materials include ceramics (e.g., PZT, AlN, and ZnO), polymers (e.g., PVDF), fibers (e.g., silk), and various composites.^[^ ^187^, ^200^ ^]^ New piezoelectric materials have also spurred the development of wearable FMG sensors. Wearable piezoelectric sensors request both good breathability for human comfort and high tensile strength for anti‐interference stability. Recently, Fan et al. introduced a 3D piezoelectric fabric strain sensor based on PVDF nanofiber and nano yarn. Its excellent permeability allowed sweat to move unidirectionally from the inner layer to the outer layer in 4 s. Besides, this 3D piezoelectric fabric fabricated through textile technology showed the highest tensile strength (46 MPa) ever reported among the reported flexible piezoelectric sensors. The excellent permeability and superior tensile strength made it promising for comfortable and long‐term wearable FMG measurements.^[^ ^201^ ^]^ In addition, researchers have explored various structural designs to enhance the sensitivity and stretchability of piezoelectric strain sensors.^[^ ^202^, ^203^, ^204^ ^]^ Among them, kirigami‐inspired patterns offer superior design versatility, enabling tailored mechanical and sensing properties across diverse application scenarios. Kirigami design refers to a structural engineering approach that introduces strategic cuts into thin piezoelectric films, thereby improving their mechanical deformability. Kim et al. developed a haptic glove incorporating multiple kirigami patterns to enable accurate detection of joint and muscle movements at the hand joints (Figure 4f). Finite element simulations showed that S1, S2, and P patterns facilitated stress distribution, whereas the H pattern concentrated stress. Experimental results revealed that the H and P patterns yielded the highest stretchability (320.8%) and sensitivity (9.86 V/cm^2^), respectively.^[^ ^205^ ^]^ Beyond passive sensor structures, active devices such as transistors can simultaneously perform signal sensing, amplification, and multiplexing.^[^ ^206^, ^207^, ^208^ ^]^ To this end, researchers have integrated piezoelectric sensors with transistor technologies to enhance measurement capability and facilitate compatibility with CMOS circuitry. For instance, Oh et al. developed a dual‐gate piezoelectric ZnO TFT on a flexible substrate with high spatial (100 µm) and temporal (10 ms) resolutions. Figure 4b shows the proposed device where the piezoelectric ZnO layer functioned as a gate switch for drain‐source current. Their 16 × 8 ZnO TFT array remained functional even when rolled over a rod with a 5 mm bending radius and sustained stable performance over 10000 bending cycles with radii ranging from 2 to 7 mm.^[^ ^207^ ^]^

Han et al. proposed an implantable piezoelectric force sensor with a 3D layout for localized FMG signal monitoring. This 3D piezoelectric microsystem comprises patterned PVDF and electrode layers capable of both energy harvesting and force sensing. 3D geometry (figure 4j) offered a higher level of functionality and extra flexibility that efficiently coupled the device with muscle contractile motions. Patterned piezoelectric energy harvesters use ultrathin PVDF serpentines that enable multidirectional and broadband vibrational energy harvesting. The devices can generate peak output RMS voltage up to 0.79 V with electrode and structural optimizations. A buckled bi‐stable design allows efficient harvesting across a wide frequency range (5–500 Hz), offering potential for powering low‐power implantable electronics from ambient vibrations or sound. Cytotoxicity assay showed that fibroblast cells cultured on PVDF (9 µm thick) and parylene‐C films exhibited no cytotoxic effects over three days, with cells maintaining normal adhesion and morphology after seven days. In vivo implantation of this 3D microsystem into the vastus muscles of a mouse (Figure 4j) successfully captured FMG signals during various movements, such as trotting and climbing.^[^ ^209^ ^]^

#### 4.1.3 Force Sensing with Capacitive Sensors

Capacitive sensing converts applied force into changes in capacitance. A capacitive force sensor typically comprises a soft dielectric layer sandwiched between two electrodes. Under mechanical stress, the dielectric layer undergoes a reduction in thickness and an isotropic increase in surface area across multiple directions. In contrast, strain sensing involves surface elongation along the direction of strain, accompanied by contraction in the transverse (width‐wise) direction.

Device microstructures, material properties, and fabrication methods have attracted researchers' interest in improving capacitive sensor performances. Dielectric and electrode layers with special structures such as wrinkled,^[^ ^210^ ^]^ mesh,^[^ ^211^ ^]^ pyramid,^[^ ^212^ ^]^ pillars,^[^ ^213^ ^]^ domes,^[^ ^214^ ^]^ and hemispheres^[^ ^215^ ^]^ were also proposed to improve sensitivity and reduce equivalent elastic modulus.^[^ ^216^ ^]^ For example, Mannsfeld et al. first proposed in 2010 a pyramid‐patterned dielectric layers, boosting sensitivity by 30 times.^[^ ^217^ ^]^ Nur et al. surpassed the theoretical limit of capacitive strain sensors’ gauge factor by introducing out‐of‐plane wrinkling deformations (Figure 4g). The proposed sensor achieved a gauge factor of 3.05 with excellent linearity (R^2^ = 0.98), durability (stable performance after 1000 times 0%–140% stretching cycles), and negligible hysteresis.^[^ ^218^ ^]^ Common electrode materials for capacitive sensing include Au, Ag, and Cu. In addition, various elastic materials like PDMS, PVDF, sponge, and Ecoflex are proposed for the dielectric core due to their low elastic modulus, enhancing the sensor sensitivity. Beyond applying microstructured interfaces, incorporating ionic hydrogels as dielectric layers significantly enhances capacitive sensor sensitivity due to their intrinsically high permittivity.^[^ ^219^, ^220^, ^221^ ^]^ Niu et al. developed a high‐sensitivity capacitive stress sensor that combined both microstructural engineering and ionic hydrogel materials. Leveraging a double interlocked microcone architecture and the supercapacitive iontronic effect provided by a PVDF‐HFP‐based ionic gel, the sensor achieved a remarkable sensitivity of 805.1 kPa^−1^. As illustrated in Figure 4c, the device featured a three‐layer configuration, where the microcone patterns mimiced the structure of human vellus hair, dermis, and hypodermis. A 4 × 4 sensor array was further integrated with electronic components to construct a glove‐mounted system for gesture morphology recognition. Real‐time gesture detection was successfully demonstrated, achieving a classification accuracy exceeding 90% based on the processed FMG signals.^[^ ^222^ ^]^ Researchers also explored new synthesis strategies for traditional materials; for instance, Xu et al. applied the Joule heating chemistry method to fabricate silicone‐based elastomers (SEs) with programmable porous structures. The proposed approach significantly reduced the fabrication time and optimized the process in a more environmentally friendly way. Due to the abundance of deformation sites in porous SEs, this porous capacitive force sensor exhibited a wide sensing range (0–500 kPa), fast response (230 ms), and good durability (over 15 000 cycles). FMG signals captured from SEs‐based sensors were successfully used for motion monitoring in a wireless human‐machine interaction system. Uneven pore distribution and inconsistent mechanical strength may become more critical in larger or more complex geometries, underscoring the need for further studies to ensure scalable fabrication and reliable mechanical performance.^[^ ^223^ ^]^

For implantable application, Lee et al. proposed a wireless 1D system with in‐vivo muscle strain sensing capability. A novel fiber‐based capacitive strain sensor was integrated with an inductive coil (Figure 4k). Two stretchable conductive fibers coated with PDMS formed the strain sensor in a double helical structure with a hollow core. The conductive fibers and hollow core served as electrodes and deformable dielectric layers, respectively. This sensor demonstrated a sensitivity around three times higher than the highest existing 2D capacitive strain sensors.^[^ ^218^ ^]^ A stable capacitive response with no significant degradation is reported after 2000 cycles of 10% strain, demonstrating its high mechanical durability. The fiber also showed excellent long‐term biocompatibility, with >65% human cardiac microvascular endothelial (HCME) cell confluency and comparable ATP levels to controls after 3 weeks in culture, indicating no cytotoxic effects. No severe inflammation or fibrosis was observed around the implantation site. As illustrated in Figure 4k, the system was implanted in a porcine leg for in vivo testing. In‐vivo implantation test demonstrated stable wireless strain sensing over 3 weeks, with minimal signal loss, exhibiting a resonant frequency shift of ≈1.45 MHz at 25.21 MHz, corresponding to ≈3.09% strain change. In the future, the device can be made more biocompatible by replacing the current Ag nanoparticles with Au nanoparticles.^[^ ^224^ ^]^

#### 4.1.4 Force Sensing with Optical Fibers

Optical fiber‐based force sensors operate by analyzing differences between the emitted and detected optical signals. The light transmitted through the optical fiber is highly sensitive to external mechanical forces, which alter its intensity, phase, or wavelength.^[^ ^225^ ^]^ Researchers have explored optical fiber force sensing through two mechanisms: Wavelength shifting and intensity attenuation. When applying a force to a fiber Bragg grating (FBG), the grating period and effective refractive index change. As a consequence, there is a wavelength shift proportional to the applied force.^[^ ^226^ ^]^ Intensity attenuation results from altered light reflection conditions due to force‐induced deformations such as stretching and bending, affecting optical path lengths and causing radiation losses.^[^ ^227^, ^228^ ^]^ Optical fiber force sensors are prized for their lightweight, electromagnetic immunity, and multiplexing capabilities, drawing significant research attention.^[^ ^229^ ^]^ Recent studies have demonstrated their integration in force monitoring across various anatomical sites, including the elbow joint,^[^ ^230^ ^]^ fingers,^[^ ^225^, ^231^ ^]^ knee,^[^ ^232^ ^]^ arm,^[^ ^233^ ^]^ and wrist,^[^ ^234^ ^]^ supporting applications in musculoskeletal assessment and human–machine interaction.

Highly stretchable optical fiber sensors have attracted significant research interest in the context of wearable applications. Zhang et al. developed a stretchable optical fiber sensor featuring a 3D helical structure, fabricated via thermal drawing. The helical geometry enabled the fiber to withstand strains up to 580% while maintaining light‐guiding capability under both stretching and bending conditions. The sensor's output power loss increased proportionally with applied strain and bending curvature, primarily due to elongated optical path length and radiation losses. Integrated into a wrist brace, the fiber effectively detected strain associated with muscle deformation during wrist movement, demonstrating its potential for real‐time biomechanical monitoring.^[^ ^234^ ^]^ Driven by the development of human‐machine interfaces and intelligent robotics, researchers also focused on the multifunctionality of optical fiber force myography sensors. Li et al. proposed a skin‐like optical fiber sensor with tactile stress and strain hybrid‐sensing capability. Four fiber Bragg grating elements were embedded between two flexible substrates (Figure 4d). Tactile and tensile sensing units were made of straight‐line and double‐x configurations, respectively, to achieve high stretchability and easy array partitioning. The sensor supports high‐fidelity multimodal detection of tactile force (up to 3.5 N), strain, and contact location, while exhibiting over 20% stretchability and achieving 92.41% accuracy in position recognition. Mounted on skeletal muscles, including flexor digitorum superficialis and flexor carpi radialis, the sensor detected strains from 1% to 20% and was used in robot‐human interaction for precise grasping operations.^[^ ^233^ ^]^ Kim et al. developed a multifunctional sensor combining heterogeneous sensing mechanisms, including optical fiber, microfluidic, and piezoresistive. Three different types of sensing principles shared the primary sensing structure without interfering with each other. The sensor layout is shown in Figure 4h, where ionic liquid functions as a microfluidic channel and a waveguide for an optical fiber. In this system, an LED and a photodiode were placed on each side of the channel to emit and record the optical signals. Silver‐plated knitted fabric integrated into the channel sidewall served as the piezoresistive sensing element. FMG signals were decoupled from complex deformation patterns using an artificial neural network. With this configuration, the researchers achieved over 95% accuracy in estimating eight deformation modes, outperforming conventional single‐mechanism approaches.^[^ ^230^ ^]^

Self‐powered FMG optical sensors are highly desirable for implantable applications. Liang et al. proposed a self‐sustaining optical sensor system that eliminates the need for external power. As illustrated in Figure 4l, the device incorporates mechanoluminescent optical fibers coated with a ZnS: Cu phosphor layer, which emits light in response to mechanical strain. The generated light was further transmitted through an elastomer optical fiber to the photodetector. In this configuration, the light intensity linearly increased with strain. The proposed optical fiber sensor demonstrated high durability, maintaining stable mechanoluminescence intensity (< 20% decrease) over 10000 stretching–releasing cycles at 50% strain. *Ex vivo* pork muscle experiments showed the performance of the implanted FMG sensor upon different muscle tissue motions.^[^ ^235^ ^]^

We have outlined recent advancements in FMG sensors, focusing on four distinct sensing mechanisms. This section highlights how materials and device design advances have contributed to the miniaturization of FMG sensors. Other research directions, such as machine learning for signal processing and motion decoding, circuit design for FMG signal extraction, and sensor placement strategies (e.g., straps and sleeves), are beyond the scope of this section but have been systematically reviewed elsewhere.^[^ ^15^, ^180^ ^]^ Despite differences in sensing mechanisms, FMG sensors share common research focuses, including high sensitivity, linearity, stability, and durability. Representative examples of both strain‐ and stress‐based sensing approaches are illustrated above for each mechanism. To enhance sensitivity in capacitive FMG sensing, researchers have explored microstructure designs, such as pyramid patterns (2010),^[^ ^217^ ^]^ 3D wrinkling deformations (2018),^[^ ^218^ ^]^ and double‐helical structures (2021),^[^ ^224^ ^]^ progressively increasing the gain factor beyond 12. An ongoing research focus is the integration of strain and stress sensing within a single device, though minimizing mutual interference remains a key challenge. Future progress in FMG will likely stem from the combined impact of new designs and advanced materials. In addition, the development of FMG sensing is being driven by applications in human–machine interfaces and intelligent robotics. Researchers have integrated artificial intelligence to improve FMG data analysis and motion control or prediction. For instance, Li et al. combined AI‐driven spatiotemporal dynamic logic analysis with optical FMG sensors to achieve human‐machine interaction control commands for multifunctional interaction. To enhance FMG sensor functionality, researchers are investigating wireless power and data transmission, multiparameter sensing, high‐density sensor arrays, and high SNR detection. Achieving these goals requires innovations such as 3D architectures, advanced CMOS chips, optimized algorithms, and improved interconnects. For implantable FMG applications, piezoelectric FMG sensing is particularly promising due to two key advantages: 1) it operates without an external power supply and 2) it can function as an energy harvester. Recently, biodegradability has gained attention among academics and clinicians, as it eliminates the need for surgical sensor removal. Researchers have developed biodegradable force sensors for implantation applications such as tendon rehabilitation, arterial blood flow monitoring, and pulse wave detection.^[^ ^236^, ^237^, ^238^ ^]^ Extending biodegradability concepts to muscle sensing represents an exciting future direction for implantable FMG research.

### 4.2 Acoustic Wave Sensing: Acoustic Myography (AMG) and Sono Myography (SMG)

Various acoustic sensing technologies have been developed for monitoring biomechanical signals generated by muscle activity. The two primary acoustic sensing modalities are acoustic myography (AMG) and sonomyography (SMG). AMG, also known as mechanomyography, phonomyography, or vibromyography, was first introduced in the 1980s.^[^ ^240^ ^]^ It captures acoustic waves produced by lateral resonance within muscle fibers during contraction.^[^ ^241^ ^]^ AMG signals typically fall within the 5 to 100 Hz range, and their time‐ and frequency‐domain features reflect the muscle physiological status.^[^ ^13^, ^242^ ^]^ The term SMG, first introduced in 2006,^[^ ^243^ ^]^ involves the use of an external source apparatus (e.g., piezoelectric transducer) to emit and detect ultrasound waves in the 2 to 14 MHz range. These waves interact with muscle tissue and are measured through reflection or transmission modes.^[^ ^244^ ^]^ Changes in the physical and physiological properties of muscle alter ultrasound signal intensity and propagation speed.^[^ ^14^ ^]^ SMG has demonstrated high spatial (0.5 mm) and temporal (25 Hz) resolution, along with a penetration depth in muscle tissue of up to 17 mm, enabling deep‐tissue analysis.^[^ ^245^, ^246^ ^]^ Both AMG and SMG utilize acoustic waves and offer several advantages: low sensitivity to variations in skin condition, immunity to electrical crosstalk noise, and non‐invasive measurement capabilities. Recent research efforts have focused on the miniaturization of both technologies to improve portability and enable their practical use in daily applications.

**Figure 5:**
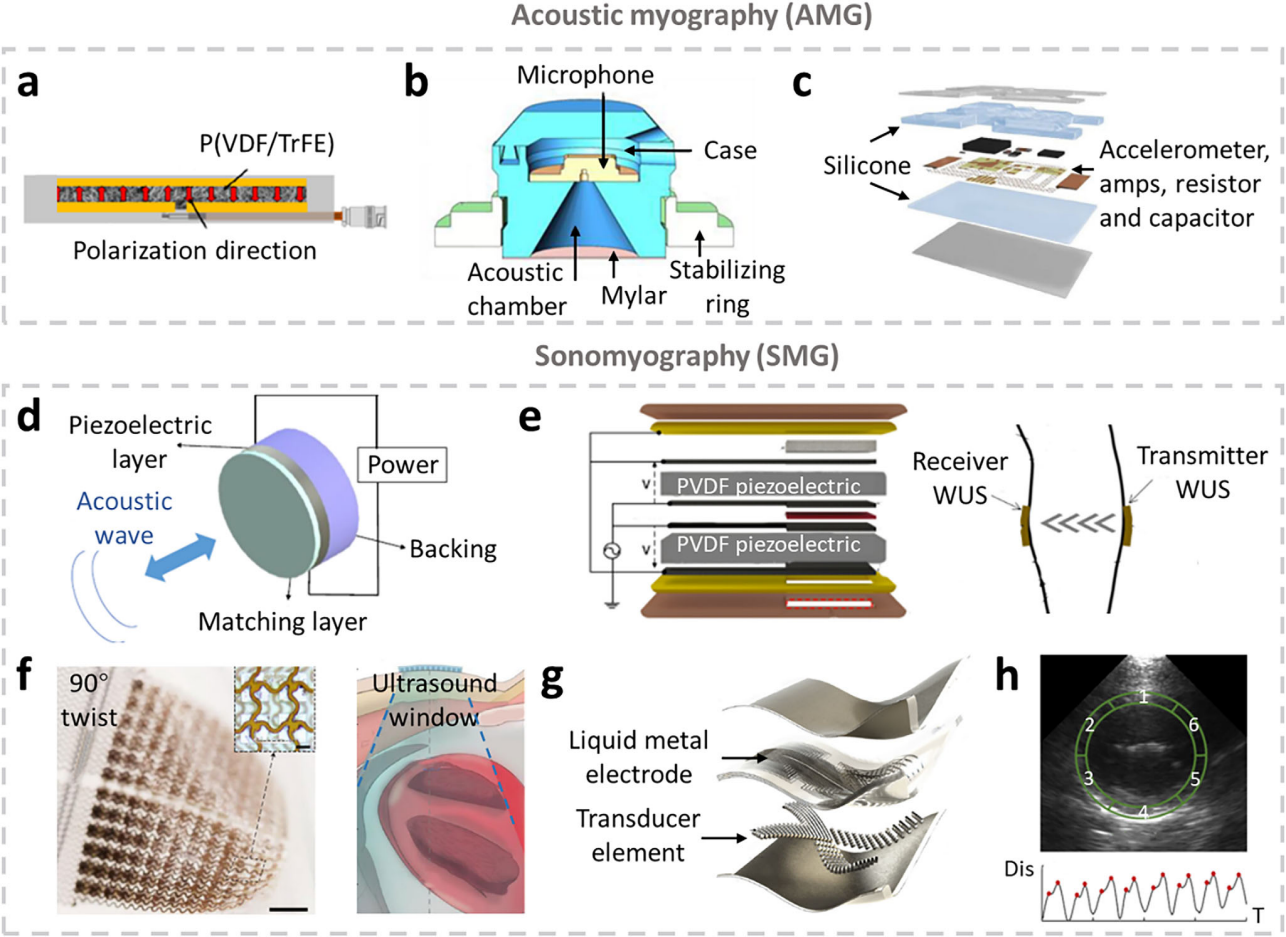
Wearable devices for Acoustic Myography (AMG) and Sono Myography (SMG) signal monitoring. a) Schematic illustration of a piezoelectric polymer nanofibers sensor based on P(VDF/TrFE) for AMG monitoring.^[^ ^250^ ^]^ b) Cross‐section view of a microphone sensing device for AMG armband application.^[^ ^248^ ^]^ c) Schematic illustration of the mechano‐acoustic sensing platform for cardiac activity detection.^[^ ^251^ ^]^ d) Schematic of an ultrasound transducer.^[^ ^257^ ^]^ e) Schematic design of a single‐element wearable ultrasonic sensor (left) and the through‐transmission (TT) mode measurement configuration of contractile parameters of the lower leg (right).^[^ ^252^ ^]^ f) The optical picture of the stretchable ultrasonic phased array (12 × 12) under 90° twist (left) and the schematic of the ultrasonographic window in cardiac activity monitoring (right). The inset shows four transducer elements. Scale bars: 2 mm (main image) and 300 µm (inset).^[^ ^255^ ^]^ g) The schematic of a wearable ultrasound imager (32 elements in each orthogonal array direction) for continuous myocardium movement recording.^[^ ^256^ ^]^ (h) The B‐mode images of the left ventricle in basal view (top) and the relationship between displacement (Dis) for segments 3 and changing time (T) (bottom).^[^ ^256^ ^]^ All pictures are reproduced with permission.

Transducers used in AMG include piezoelectric sensors,^[^ ^247^ ^]^ microphones,^[^ ^248^ ^]^ and accelerometers.^[^ ^249^ ^]^ Researchers have built wearable AMG sensors based on the above three mechanisms, respectively. For example, Abe et al. developed a wearable sensor using piezoelectric polymer poly(vinylidene fluoride trifluoroethylene) copolymer (P(VDF/TrFE)) nanofibers for AMG measurement during exercise (**Figure** **5**a). The piezoelectric nanofibers were fabricated through electrospinning with optimized concentration, thickness and electric field. With this wearable sensor, researchers successfully detected the force signals from the rectus femoris muscle.^[^ ^250^ ^]^ Sebastian et al. proposed an armband integrating four microphone sensors for force measurement. One sensor integrated a microphone (Knowles SPU1410LR5H‐QB) with an acoustic resonating chamber closed by mylar membrane (Figure 5b). The AMG signal excited the mylar film, altering the air pressure within the chamber, which was then detected by the microphone. The armband, including four sensors positioned at different locations, achieved gesture classification.^[^ ^248^ ^]^ Liu et al. developed a mechano‐acoustic‐electrophysiological sensing platform with minimal mass (213.6 mg) and low effective Young's moduli (31.8 kPa in x direction and 31.1 kPa in y direction) for monitoring heart physiological signals. This platform simultaneously captured acoustic waves from heart muscle contractions and electrophysiological signals using accelerometers and capacitive electrodes. The accelerometers and capacitive electrodes were mounted together with the acquisition electronics on a flexible PCB (Figure 5c). On‐device edge computing included low‐pass (500 Hz) and high‐pass (15 Hz) filters and preamplification, supporting edge‐level noise reduction and signal conditioning before transmission. In this study, the researchers showed a close correlation between the AMG and electrophysiological signals from the heart, offering several insights into cardiomyocyte contraction activities.^[^ ^251^ ^]^

Researchers have employed two measuring configurations for SMG: pulse‐echo (PE) mode and through‐transmission (TT) mode. In PE mode (Figure 5d), the emitter and receiver are the same transducer. Conversely, TT mode (Figure 5e) utilizes separate emitters and receivers on either side of the muscle. PE mode is predominantly used in SMG, although TT mode may lead to higher SNR due to shorter travel distances in the tissue.^[^ ^252^ ^]^ SMG technology is also categorized based on the type of information generated. The most used ones are A‐mode (amplitude mode), B‐mode (2D mode), and M‐mode (motion mode). A‐mode offers data in one dimension where the y‐axis is tissue depth, and the x‐axis is echoing amplitude. B‐mode provides a 2D image of the measured tissue by scanning a few hundred transducer elements simultaneously. The brightness of the image reflects the echo amplitude. M‐mode generates a video of the scanned 2D tissue areas based on the same working principle as B‐mode.^[^ ^253^, ^254^ ^]^

The TT configuration has an advantage for measuring thick muscle tissue (e.g., arm and leg), as its ultrasound travelling distance is 50% shorter compared with the PE configuration. AlMohimeed et al. developed a TT‐mode AMG sensor with a single‐sensing element where the emitter and receiver were placed at two sides of the tested muscle. The device collected amplitude information and measured the skeletal muscle contractile parameters. The proposed emitter comprised a double‐layer structure where two PVDF layers with antiparallel polarization were bonded together (Figure 5e). This structure effectively increased the amplitude of emitting ultrasonic waves and reduced the value of the applied alternative voltage. Various contractile parameters (e.g., contraction time and thickness) in the human lower leg were successfully extracted from the SMG signal.^[^ ^252^ ^]^ Owing to the planar layout of PE configuration where the emitter and receiver were the same transducers, researchers have integrated multiple sensing elements into a single AMG sensing system to achieve higher functionality. In 2021, Wang et al. developed a stretchable ultrasonic sensor with 12 × 12 phased arrays with 0.8 mm pitch and 16% biaxial elastic stretchability, enabling conformal integration on skin (figure 5f). Each element operated at ∼2 MHz with a 770 µm pitch, matching the ultrasonic wavelength to enhance beam convergence and reduce phase aberration, while delivering 0.47 W/cm^2^ acoustic intensity safely. The phased‐array structure allowed steering of beamforming waves from ‐20° to 20°. The formed beam effectively increased the SNR of the sensor, which was maintained at ≈20 dB under mechanical deformation. The beamforming remained reliable under 20% stretching or 20% compression. The stretchable device showed great durability with minimal performance degradation after 60 days of repetitive use. The ultrasonographic image generated with this device showed accurate real‐time imaging of the myocardium movement at different depths.^[^ ^255^ ^]^ Later, in 2023, Hu et al. from the same group proposed a new wearable ultrasound imager that achieved continuous M‐mode and B‐mode myocardium image recording. In this system, each transducer element comprised a 1–3 piezoelectric composite layer and a backing layer. A novel orthogonal configuration (32 × 32 elements) was applied to achieve a comprehensive view of the heart without manual rotation (Figure 5g). The device comprised screen‐printed liquid metal electrodes on the elastomer substrate, offering mechanical compliance with up to 110% stretchability. The structure information of the left ventricle, including myocardial thickness and displacement, was successfully captured (Figure 5h). The displacement of myocardium boundaries in segment 3 was recorded in M‐mode, highlighting the two adjacent peaks (red dots) representing the relaxation of cardiomyocytes. The main limitations of this research include the reliance on a flexible cable connection for data transmission and processing, which hinders full system miniaturization and untethered operation.^[^ ^256^ ^]^

AMG and SMG can be understood as passive and active approaches, respectively, to capturing muscle biomechanical signals through acoustic sensing. This section discussed AMG based on three mechanisms (piezoelectric sensors, microphones, and accelerometers) and SMG in two configurations (TT mode and PE mode). Representative examples illustrate how sensor miniaturization enhances both accessibility and performance. Other research directions, such as feature extraction algorithms, artificial intelligence for movement classification, dynamic reliability, and probe placement/orientation, are beyond the scope of this review. In AMG, the detected acoustic waves originate from muscle fiber vibrations, but these signals undergo significant attenuation before reaching the sensing unit. Therefore, improving SNR through both hardware and software remains a critical challenge. In SMG, TT mode is more commonly used in clinical applications due to its higher accuracy and deeper penetration depth. In contrast, PE mode is preferred for daily wearable applications because its planar configuration facilitates miniaturization and integration of additional components, such as data processing and power transmission units.^[^ ^252^ ^]^ Further research should focus on enhancing in situ image acquisition and processing capabilities in wearable SMG systems, essential for improving real‐time muscle monitoring and functional assessment.

### 4.3 Electrical Impedance Myography (EIM)

Electrical impedance tomography (EIT), also known as electrical impedance myography (EIM) in muscle activity evaluation, was initially developed by Henderson and Webster in the 1970s.^[^ ^258^ ^]^ It is a non‐invasive technology to assess the impedance distribution within the muscle tissue. EIM involves applying an alternative current in the microampere range at frequencies ranging from 1 kHz to 1 MHz to an electrode pair attached to the tissue or skin surface. The resulting voltages across other electrode pairs are measured to capture the EIM signal.^[^ ^259^, ^260^ ^]^ The tissue under measurement can be modelled as an equivalent electric circuit comprising resistors and capacitors, representing the connective muscle tissue and myofiber membranes, respectively. The electrical properties derived from the measured voltages, such as resistance, reactance, and phase angle, reflect the structural characteristics of muscle tissue.^[^ ^259^ ^]^ EIM measurements utilize three main driving modes: adjacent, opposite, and trigonometric. In the adjacent and opposite modes, electrode pairs are separated for current injection and voltage measurement. In contrast, the trigonometric method employs the same electrodes for both functions.^[^ ^261^ ^]^ Reconstruction of an EIM measurement occurs after collecting voltages from all electrode pairs, revealing the impedance distribution across the muscle tissue area. Extensive research currently focuses on developing algorithms and utilizing artificial intelligence to achieve rapid and accurate image reconstruction.^[^ ^262^, ^263^ ^]^ EIM is applied in hand gesture recognition and human‐machine interfaces.^[^ ^264^, ^265^, ^266^, ^267^ ^]^ A limited amount of research focuses on miniaturizing EIM sensing for small‐scale wearable applications. Efforts to scale down EIM systems include EIM‐based artificial skin. Most EIM‐based artificial skin is made of conductive rubber and conductive fabric.^[^ ^261^, ^268^, ^269^ ^]^

## 5 Muscle Oxygenation Monitoring

**Figure 6:**
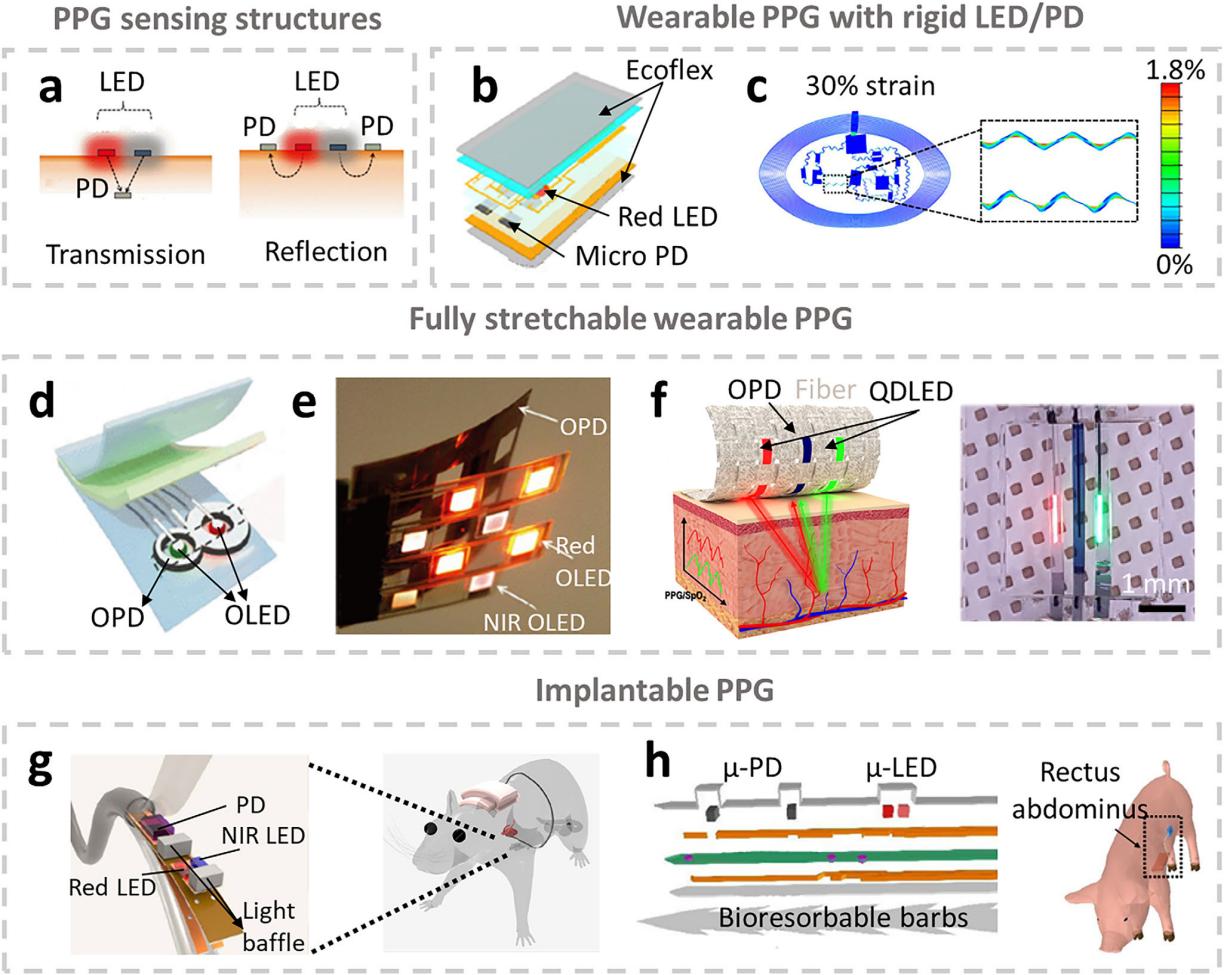
Wearable and implantable sensors for Photoplethysmography (PPG) signal monitoring. a) PPG measurement can be performed in two modes: transmission mode and reflection mode. Reproduced with permission.^[^ ^296^ ^]^ b) Schematic illustration of a reflective PPG device where a rigid light‐emitting diode (LED) and rigid micro‐photodiode are integrated on a thin Ecoflex substrate.^[^ ^294^ ^]^ c) Finite element analysis of the strain in PPG sensors interfaced with other electronics through serpentine interconnections, with an applied uniaxial strain of 30%.^[^ ^295^ ^]^ d) A fully organic PPG sensing patch where an 8‐shape photodiode surrounds red and green LEDs.^[^ ^286^ ^]^ e) A four‐by‐four fully organic PPG oximeter array.^[^ ^287^ ^]^ f) Schematic illustration (left) and optical picture (right) of quantum‐dot‐based pulse oximetry on fiber substrate.^[^ ^283^ ^]^ g) An implantable catheter‐type PPG oximeter for cardiac oxygen saturation measurement (left) and the 3D schematic illustration of the catheter implanted in a rat heart in vivo and wireless module placed on the back (right).^[^ ^297^ ^]^ h) An implantable probe featuring biodegradable barbs, PPG sensing units (left), and the 3D picture illustrating the probe implanted in porcine rectus abdominus in vivo (right).^[^ ^277^ ^]^ All pictures are reproduced with permission.

**Figure 7:**
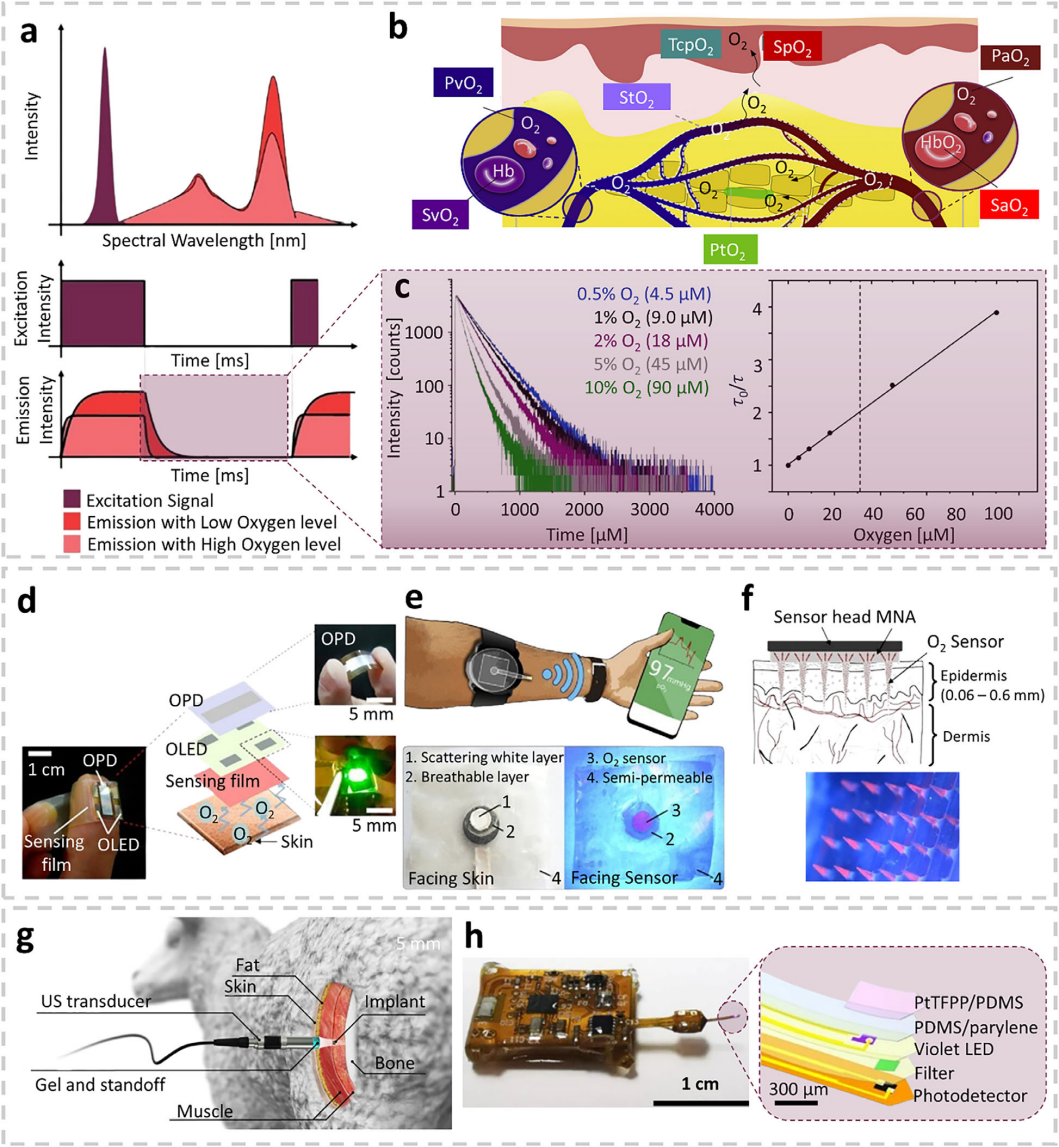
Continuous muscle tissue oxygenation monitoring via light quenching. a) Graphical representation of typical luminescence intensity (up) and lifetime (bottom) recordings.^[^ ^342^ ^]^ b) Comparison of modalities for clinical and non‐clinical muscle tissue O_2_ evaluation; PaO_2_: Arterial Oxygen Partial Pressure [mmHg], which reflects the quantity of unbound oxygen molecules that are freely dissolved in arterial plasma, PtO_2_: Tissue Oxygen Partial Pressure [mmHg], PvO_2_: Venous Oxygen Partial Pressure [mmHg], SaO_2_: Arterial Hemoglobin Oxygen Saturation [%] which refers to the ratio of oxygenated hemoglobin to the total hemoglobin in arterial, SpO_2_: Peripheral Oxygen Saturation [%], StO_2_: Tissue Oxygen Saturation [%], SvO_2_: Venous Hemoglobin Oxygen Saturation [%], TcpO_2_: Transcutaneous Oxygen Tension [mmHg].^[^ ^377^ ^]^ c) Phosphorescence decay curve (left) and corresponding Stern‐Volmer plot (right) for a 5 w/v% silk‐PdBMAP film in PBS at 37 °C. The dotted line on the Stern‐Volmer plot indicates the oxygen concentration at which the phosphorescence lifetime is reduced to 50% of its value in the absence of oxygen.^[^ ^376^ ^]^ d) A wearable oxygen sensor includes a sensing film, an organic photodiode (OPD), and organic light‐emitting diodes (OLEDs) mounted on a human thumb.^[^ ^351^ ^]^ e) An optical wireless wearable system for non‐invasive TcpO_2_ monitoring.^[^ ^354^ ^]^ f) Phosphorescent microneedle array for minimally invasive pO_2_ measurement before insertion.^[^ ^362^ ^]^ g) Schematic of an implanted system for local tissue oxygen monitoring in sheep, where a free‐floating wireless oxygen implant is surgically positioned. An external ultrasound (US) transducer establishes a wireless acoustic link with the implant.^[^ ^366^ ^]^ h) Image of the oxygen‐sensing implant connected to the battery‐free circuit, featuring (from top to bottom) a PtTFPP/PDMS sensing film, a PDMS/parylene encapsulation layer, an InGaN violet LED, a filter, and an InGaP photodetector on a polyimide (PI) substrate.^[^ ^367^ ^]^ All pictures are reproduced with permission.

Continuous oxygen monitoring plays an important role in physiological monitoring, not only uncovering cellular energy metabolism and sustaining muscle tissue regeneration but also enabling real‑time detection of transient hypoxemic episodes that intermittent assessments miss.^[^ ^270^, ^271^ ^]^ Continuous monitoring of interstitial pO_2_ within muscle tissue enables real‐time tracking of oxygen fluctuations caused by ischemia, mechanical trauma, or postoperative recovery. This capability enables oxygen regulation to prevent metabolic imbalances and support tissue repair by promoting angiogenesis, collagen synthesis, and cell growth.^[^ ^270^, ^271^, ^272^, ^273^, ^274^, ^275^, ^276^, ^277^ ^]^ Consequently, monitoring of muscle tissue oxygen levels is relevant in managing a range of medical conditions, such as ischemia‐reperfusion injury, organ transplantation, plastic and reconstructive surgery, diabetic and pressure ulcers, traumatic or surgical wounds, cardiac tissue restoration, and neural regeneration.^[^ ^272^, ^273^, ^274^, ^275^, ^276^, ^277^ ^]^ This section explores recent advancements in oxygen‐sensing technologies tailored for muscle tissue monitoring. We highlight the latest developments in wearable and implantable sensors, focusing on methods that leverage absorption‐based techniques, such as photoplethysmography (PPG, **Figure** **6**), electrochemical oxygen sensors, and luminescence‐based oxygen quenching (**Figure** **7**).

### 5.1 Light Absorption‐Based Indirect Muscle O_2_ Sensing: Photoplethysmography (PPG)

Tissue oxygenation can be assessed indirectly by measuring tissue oxygen saturation (StO_2_) or peripheral oxygen saturation (SpO_2_), both of which rely on the absorption contrast of hemoglobin when bound to oxygen. This principle is utilized in spectroscopic techniques such as near‐infrared spectroscopy (NIRS), commonly used in surgical and ICU settings. For bedside monitoring and ambulatory healthcare applications, pulse oximetry systems based on photoplethysmography (PPG) are widely employed. PPG is particularly significant in assessing muscle vitality, as continuous aerobic metabolism depends on an adequate oxygen supply. By monitoring key physiological parameters like oxygenation,^[^ ^278^ ^]^ heart rate,^[^ ^279^ ^]^ and blood pressure,^[^ ^280^ ^]^ PPG enables real‐time assessment of circulatory function. There are two primary PPG measurement modes: transmission and reflection. In transmission mode (Figure 6a), the light source and detector are positioned opposite to each other, typically ideal for fingertip testing. Alternatively, reflection mode offers advantages in device integration and flexibility in placement. This mode is primarily utilized for continuous monitoring with wearable and implantable PPG devices.^[^ ^281^ ^]^

Originally invented by Alrick in 1937,^[^ ^282^ ^]^ PPG technology has seen rapid advancements, including improvements in materials,^[^ ^283^, ^284^, ^285^ ^]^ device structure,^[^ ^286^, ^287^, ^288^, ^289^ ^]^ readout circuits,^[^ ^290^, ^291^ ^]^ and ultra‐low‐power, low‐noise CMOS image sensors.^[^ ^292^, ^293^ ^]^ It is now widely used in clinical and ambulatory settings, especially in surgical and interventional procedures, where real‐time monitoring of heart rate and SpO_2_ helps detect early signs of cardiovascular instability. Beyond clinical applications, PPG has been seamlessly integrated into consumer wearables such as smartwatches, rings, baby socks, and earbuds, allowing for continuous health tracking. These devices can also analyze blood pressure trends, heart rate variability, and sleep patterns based on PPG data.^[^ ^280^ ^]^ Moreover, smart patches embedded with PPG sensors are being developed to monitor oxygen levels in localized muscle tissue, aiding in rehabilitation, sports science, and chronic disease management.^[^ ^283^, ^284^, ^285^, ^286^, ^287^, ^288^, ^289^, ^294^, ^295^, ^296^ ^]^ Furthermore, implantable PPG devices provide valuable high‐resolution spatiotemporal insights into localized oxygenation in muscle tissue, further enhancing the ability to track and understand physiological and pathological processes in real‐time.^[^ ^277^, ^297^, ^298^, ^299^ ^]^

#### 5.1.1 Wearable Photoplethysmography (PPG)

To advance the capabilities of wearable photoplethysmography (PPG) technology, recent innovations have focused on developing ultra‐flexible, skin‐conformal devices that closely adapt to body contours, ensuring high‐fidelity signal acquisition while maximizing user comfort during long‐term monitoring (**Table** **4**).

**Table 4:** Summary of structural features and key performance metrics of recent wearable and implantable Photoplethysmography (PPG) sensors.

Category	Light source			Light detector			Source‐detector distance	Layout Power Ref		
	Emission Peak	Property	Material	Response Range	Property	Material				
Inorganic LED or PD wearable	625 nm; 950 nm	NA	SFH 4043 and LR QH9F	NA	NA	EMD7000 × 01	2 mm	2 LED 1 PD	1‐3 mA	[301]
Inorganic LED or PD wearable	620 nm	E: 1.5 mW/cm^2^	n‐GaAs	NA	R is 0.233 A/W at zero bias	GaAs	200 µm and 700 u m	1 LED 4 PD	NA	[294]
Inorganic wearable (stretchable structure)	640 nm; 940 nm	NA	Commercially available	NA	NA	Commercials available	500 um	2 LED 1 PD	5 mA as peak current	[95]
Inorganic wearable (stretchable structure)	690 nm; 830 nm	NA	SFH 4043 and LR QH9F	NA	NA	EMD7000 × 01	6 mm	2 LED 1 PD	15 mW	[295]
Inorganic wearable (stretchable structure)	680 nm; 900 nm	NA	AS7341 ams‐OSRAM AG	480‐900nm	R is high in 450‐750 and 850‐1000	L130‐6580002011001 and SFH 4043	3‐13.5 mm	4 LED and 4 PD	NA	[302]
Fully stretchable wearable	612 nm; 725 nm	EQE: 2‐10%	Semiconducting polymers	400‐850nm	The average EQE in 400‐850nm is 30%	OPD active layer	0.5 cm; 0.8 cm; and 1.1 cm	8 LED and 8 PD	0.1 mA. cm^−2^	[287]
Fully stretchable wearable	536 nm; 631 nm	Current efficiency: 61.22 and 79.28 mW/A for red and green	CdSe/ZnS QD layer	NA	R are 0.298 and 0.291 A/W for 631 and 536 nm	PBDBT‐2F/IT‐4F	0.3 mm	2 LED and 1 PD	0.061 mW	[283]
Fully stretchable wearable	520 nm; 610 nm	EQE: 21.9% and 20.8% for green and red	TCTA:Ir(ppy)_3_ and NPB:Ir(MDQ)_2_acac:B3PYMPM	NA	R: 0.29 A/W @520 nm R: 0. A W^−1^ @610 nm	C70/TAPC	0.6 mm	2 LED and 1 PD	0.048 mW	[286]
Implantable	645 nm; 950 nm	E: 14.4 mW mm^−2^	Commercial LED	S (λ) > 10%, 400–1100 nm	Photocurrent response time<1 ms	EMD7000 × 01	2 mm	2 LED and 1 PD	1.8 mA ‐ 3.1 mA	[297]
Implantable	660 nm; 850 nm	EQE are 25% and 20% for 660 and 850 nm	TCSD14‐660 and TCSD14‐850	S (λ) > 20%, 400‐1100 nm	NA	T1197P6	4mm and 7mm	2 LED and 2 PD	6 mW ‐ 15 mW	[277]

PPG, photoplethysmography; S (λ), relative spectral sensitivity; R, responsivity; E, irradiance; NFC, near‐field communication; LED, light emitting diode; PD, Photodiodo; OPD, organic photodiode; NIR, near infrared; EQE, external quantum efficency; PEN, polyethylene naphthalate; PEDOT:PSS, poly(3,4‐ethylenedioxythiophene) polystyrene sulfonate; P3HT/PCBM, poly(3‐hexylthiophene)/(6,6)‐phenyl‐C61‐butyric acid methyl ester; C70/TAPC, C70/4,4′‐cyclohexylidenebis[N,N‐bis(4‐ methylphenyl)benzenamine]; NA, not available.

Conventional wearable PPG systems utilize rigid inorganic LEDs and photodetectors (PDs) as sensing elements, which result in a mechanical mismatch with the soft, deformable nature of human skin. To address this issue and improve signal stability during motion, recent research has focused on integrating flexible encapsulation materials, such as polyimide, silicone elastomers, and other stretchable organic substrates, that promote better skin conformity, enhance adhesion, and reduce motion‐induced artifacts during continuous physiological monitoring.^[^ ^294^, ^300^, ^301^ ^]^ For example, Kim et al. utilized silicone elastomer (Ecoflex) to create a thin, flexible package with a low elastic modulus of 30 kPa, lower than the typical 100 to 1000 kPa range for human skin, ensuring both measurement stability and flexibility (Figure 6b). The electrical performance of their device showed no significant degradation after 1000 cycles with a 3 mm radius bending test.^[^ ^294^ ^]^ In addition to improving mechanical compatibility, researchers have focused on miniaturizing device sizes while simultaneously increasing their functionality. Kim et al. developed a thin flexible PPG system with a diameter of 10 mm, a thickness of 0.9 mm, flexural rigidity of 8.7 × 10^−2^ Nm, and the ability to bend to a radius of 3 mm, with wireless powering and communication, supporting long‐term continuous operation.^[^ ^301^ ^]^

In addition to relying on the inherent flexibility of substrates and encapsulation materials, stretchable structures are introduced to enhance shape adaptability further, allowing wearable PPG devices to conform more closely to the skin contours during various movements, thereby reducing motion artifacts and improving the SNR. These techniques include serpentine interconnections,^[^ ^95^, ^295^, ^302^ ^]^ watch‐chain interconnections,^[^ ^300^ ^]^ and island‐bridge structures.^[^ ^303^ ^]^ The light intensity is sensitive to external condition changes, including the distance between the light emitter and detector. Therefore, it is crucial to stabilize the distribution of rigid optoelectronic components during skin deformation. Li et al. introduced an all‐in‐one wearable PPG system where an island‐bridge structure effectively isolated strain from the functional components. Their numerical and experimental analysis verified the light path stability and the structure integrity under significant strain (35% tensile strain), with minimal deformation observed at the device center.^[^ ^303^ ^]^ Kim et al. proposed a wireless epidermal PPG fabricated with serpentine copper interconnections to isolate electronic components from strain and increase its stretchability. Their finite element simulations confirmed that strain was predominantly distributed along the serpentine interconnections, ensuring proper functioning of PPG devices even with 30% stretching (Figure 6c). The maximum principal strain in copper was 1.8% under 30% stretching, which was much lower than its fracture strain of 5%.^[^ ^295^ ^]^ The serpentine shape is widely used to enhance stretchability; however, it increases the overall length, leading to power consumption and signal attenuation. Li et al. implemented watch‐chain metal (Cu/Cr) interconnections to improve both mechanical and electrical properties of PPG sensors. Devices utilizing watch‐chain patterns demonstrated a maximum strain capability 40% higher than the serpentine‐based designs and 27% lower impedance.^[^ ^300^ ^]^ Neonates in the intensive care unit (ICU) are highly vulnerable, and their delicate, underdeveloped skin requires specialized wearable PPG devices designed with low‐profile, flexible, and skin‐conformable features to ensure comfort and accurate monitoring. These devices are optimized to minimize pressure and friction while being sensitive enough to detect subtle changes. Chung et al. combined serpentine interconnections with microfluidic chambers filled with nontoxic ionic liquid to achieve enhanced mechanical isolation between the interconnected rigid electronics components and the skin. The microfluidic channel design effectively reduced the effective modulus, resulting in 2.5 times lower stresses compared to similar designs without microfluidics.^[^ ^95^ ^]^

While inorganic materials are widely recognized for their superior photoelectric properties, there is a growing interest in incorporating intrinsically stretchable optoelectronic devices into fully stretchable systems.^[^ ^304^ ^]^ This is because, while inorganic devices offer high performance in terms of resolution, organic devices provide greater flexibility and integration potential, essential for wearable and skin‐conformable PPG systems. By integrating stretchable optoelectronic devices, such as quantum dot (QD) LEDs and QD PDs,^[^ ^283^, ^284^, ^285^ ^]^ along with organic LEDs (OLED) and organic photodiodes (OPD),^[^ ^286^, ^287^, ^288^, ^289^ ^]^ researchers aim to combine the advantages, enhancing the overall performance, flexibility, and adaptability of PPG systems without compromising SNR and resolution.

The geometric arrangement of OLEDs and OPDs is critical for minimizing noise in PPG signals. So far, researchers have proposed various geometries, including circular,^[^ ^286^, ^288^, ^289^ ^]^ bracket,^[^ ^288^ ^]^ and rectangular configurations.^[^ ^287^, ^288^ ^]^ Khan et al compared the three introduced designs, highlighting the superiority of circular structures in maximizing PPG signal magnitude when the OLED surrounded the OPD. Using the circular geometry, they observed 48.6% signal magnitude improvements in the red channel compared to the rectangular geometry.^[^ ^288^ ^]^ While less efficient, rectangular designs remained popular, particularly in configurations where the LED and PD are placed side by side. To achieve high SNR and low power consumption for long‐term wearable applications, Lee et al. proposed a more advanced circular configuration. The ideal system structure showed an OPD wraparound layout where the OPD resembles the “number 8” around small circular OLEDs for each red and green emission (Figure 6d). Geometric dimension parameters such as ring width and radius played a significant role in influencing PPG performance and achieved optimal SNR≈40. The system can operate at as low as a few tens of microwatts, which is far less than the power typically required for the inorganic counterparts (the order of milliwatts).^[^ ^286^ ^]^ Later, Bilgaiyan et al. looked into more detailed optimizations of circular OLED/OPD configuration. They tested the power consumption and signal amplitudes of various circular wrapping geometries of OLEDs and OPDs. Their findings revealed that a circular OLED surrounded by a circular OPD resulted in lower power consumption and higher PPG signal magnitude compared to the OPD surrounded by OLED. They identified limited photon accessibility from the OLED to the OPD as a potential reason for the suboptimal performance observed in the OLED wrapping structure.^[^ ^289^ ^]^ In addition to geometric shape, the emitter‐detector distance also significantly influenced PPG performance; shorter distances improve signal quality by reducing photon loss. However, overly short distances can lead to signal saturation, potentially due to direct coupling between the LED and PD, a problem that might be mitigated by adding an optical barrier between them. As most organic PPG sensors are based on single‐point measurements, it is difficult to have a comprehensive understanding of large‐area muscle tissue oxygenation. Khan et al. devised a matrix design in flexible PPG sensing, which opened a new avenue to further improve the sensing performance. The device featured multiple OLEDs and OPDs (Figure 6e), aiming to surround the detector with multiple light sources to reduce photon loss and enable 2D oxygenation mapping. They observed a mean error of 1.1% between detected oxygen saturation using the commercial probe and the proposed PPG matrix. The main limitations of this study include its reliance on specific body locations, like the forehead, for optimal pulsatile signal detection. Additionally, broader validation across diverse clinical scenarios and body types is still needed to establish general applicability.^[^ ^287^ ^]^

Due to their ultrathin thickness, in the tens of nanometers range, nanomaterials like QD active layers offer an excellent opportunity for creating stretchable LEDs and PDs. Although organic optoelectronic components showed form‐factor advantage, their broad full width at half maximum (FWHM) and vulnerability to moisture still limit their accuracy and long‐term medical application. Lee et al. integrated a QD LED into a fiber‐based PPG system, which can be woven into actual wearable clothes (Figure 6f). The QD LED utilized a 20 nm CdSe/ZnS QD material as its light‐emitting active layer, while the OPD was based on active organic materials. The QD LED exhibited a narrow FWHM of ≈30 nm, ensuring highly efficient light emission. These functional components were mounted on a fiber substrate, which allowed for precise oxygenation measurements with waveguide mode‐free characteristics. The system demonstrated good flexibility, maintaining functionality up to 1.3% strain after 1000 bending cycles.^[^ ^283^ ^]^ Kim et al. proposed QD PPG with wrinkled structure achieved by pre‐stretching the substrate and transfer printing of ultrathin QD optoelectronics. The foldable form led to further enhancement of stretchability. The QD LED and QD PD continued to operate even under high strain conditions, with characteristics remaining unchanged up to 70% strain for the LED and 40% strain for the PD.^[^ ^284^ ^]^ Nisar et al. proposed a chemically modulated WSe_2_ FET photodetector, based on 2D nanomaterials, that exhibited high photoresponsivity of 241 mA/W and fast response times (rise: 8 ms; fall: 18 ms), making them highly suitable for detecting subtle optical signals in PPG‐based muscle oxygenation systems. Given their flexibility and skin‐conformability features, these devices represent a promising next‐generation photodetector platform for PPG sensing.^[^ ^305^ ^]^

#### 5.1.2 Implantable Photoplethysmography (PPG)

Implantable PPG devices have been developed to monitor localized O_2_ saturation within deep tissues in vivo, enabling signal acquisition from deeper layers without interference from superficial skin or surrounding tissues.^[^ ^306^ ^]^ One key advantage of implantable PPG is the ability to extend the interoptode distance, the spatial separation between the light source and detector. In wearable systems, increasing this distance typically leads to significant light attenuation. However, implantable configurations largely mitigate this effect, making it feasible to increase the interoptode distance to probe a larger tissue volume and enhance signal sensitivity. This design trade‐off, between increased probed volume and greater attenuation, requires careful optimization to ensure reliable and accurate signal acquisition.^[^ ^301^, ^307^ ^]^ Overall, implantable PPG systems address the sensitivity limitations of wearable devices by supporting greater interoptode spacing, thereby enabling more precise and robust oxygenation monitoring.

Researchers have conducted a variety of in vivo animal tests using different implantable PPG technologies, targeting applications in diverse tissues such as myocardium,^[^ ^297^ ^]^ rectus abdominis,^[^ ^277^ ^]^ brain,^[^ ^298^, ^299^ ^]^ and intestine.^[^ ^308^ ^]^ Lu et al. pioneered a catheter‐tube‐type implantable PPG sensor for real‐time cardiac oxygenation measurement (Figure 6g). The oximeter was powered by a 45 mAh rechargeable lithium‐ion battery housed in a flexible, skin‐mounted module, enabling at least 22 hours of continuous operation. Data was transmitted in real time and stored via a smartphone‐based Graphical User Interface (GUI), supporting continuous monitoring. Wireless communication was achieved using Bluetooth Low Energy (BLE), allowing untethered data transmission from the implant to external devices. Adjustable optical irradiance (0–50 mW mm^−^^2^) allowed for controlled penetration depth (0–7 mm), making measuring oxygenation in both deep and superficial myocardial tissues feasible. The sensor demonstrated robust long‐term stability, showing no significant performance degradation after 10000 cycles of bending tests at a radius of 5 mm and 8 weeks of immersion in PBS. A 30‐day mouse in vivo study showed no organ damage or systemic toxicity, with blood counts and blood chemistry remaining comparable to controls, confirming good biocompatibility of the implantable oximeter. In vivo testing in a rodent model confirmed highly correlated oxygenation measurements compared to a commercial analyzer.^[^ ^297^ ^]^ In 2022, Guo et al. introduced an implantable PPG sensing probe featuring biodegradable barbs distributed along its length (Figure 6h). The biodegradable barbs, composed of poly(lactic‐co‐glycolic acid) (PLGA), enhanced device anchorage within the tissue. The biodegradable nature of the barbs allowed for easy removal of the probe from muscle tissue post‐resorption. The probe demonstrated stable functionality after four weeks of PBS immersion at 37 °C, indicating strong encapsulation and resistance to biofluid penetration. In a porcine flap model, the probe was inserted into the rectus abdominis flap to measure localized in vivo StO_2_. The measured StO_2_ changes were consistent with those obtained from a commercially available skin‐mounted StO_2_ sensor (ViOptix). Stable operation was observed in an over 1 month in vivo study, confirming its potential for long‐term tissue oxygenation monitoring. A 6‐week in vivo study in rat models showed no systemic toxicity from optical probes. There were no significant differences in body and major organ weights, blood chemistry, or complete blood counts compared to control groups. Histological analyses confirmed no organ damage, with only minor localized inflammation and fibrosis at the implantation sites. However, limitations remain in the lack of wireless charging, which restricts fully implanted long‐term use, and potential motion‐induced artifacts that could affect measurement accuracy.^[^ ^277^ ^]^

Ensuring intimate skin conformability is essential for accurate signal acquisition and user comfort in wearable PPG sensing systems. This section reviews recent developments in PPG technology, with a focus on evolving form factors, from rigid sensing units (e.g., based on Si and GaAs) on flexible substrates, to rigid components integrated with stretchable substrates, and ultimately to intrinsically flexible sensing systems (e.g., based on organic materials and nanomaterials). This evolution highlights how novel materials and structural designs have enhanced SNR, comfort, and durability in wearable PPG sensing. Beyond the shift from inorganic to organic active materials, researchers have explored eco‐friendly, self‐repairing, and energy‐harvesting materials for PPG applications.^[^ ^309^, ^310^ ^]^ Future material research will focus on improving performance while reducing costs and environmental impact. Recent PPG development trends can also be illustrated from other aspects, including fabrication process, low‐power/self‐power, signal processing, and analysis. Since SNR is a critical factor in PPG sensing, scientists are working to mitigate noise sources such as motion artifacts, pressure disturbances, skin pigmentation variations, sensor placement, and temperature fluctuations. Achieving accurate PPG measurements also requires optimizing the photoelectric characteristics of LEDs and PDs. Current research focuses on quantum efficiency, irradiation intensity, spectral sensitivity, and responsivity, all of which contribute to higher sensitivity and efficiency in PPG monitoring. In addition to advantages such as lightweight design, stretchability, and conformability, organic PPG systems can operate at the microwatt level, significantly reducing power consumption compared to inorganic counterparts. This enables longer operation times with lighter, smaller batteries. However, long‐term ambient stability remains a challenge for organic PPG sensors. Future research should focus on developing encapsulation techniques and interface materials that enhance stability without compromising functionality. Unlike patch‐type organic PPG sensors implemented on planar substrates, nanomaterials such as quantum dot layers and 1D fibers can be woven into 3D textile substrates, enabling PPG integration into clothing for improved breathability and comfort. Additionally, motion artifacts and skin pigmentation variability represent two critical challenges in real‐world applications of PPG sensing. To mitigate motion‐induced noise, advanced devices incorporate accelerometer‐assisted algorithms and adaptive filtering techniques that isolate true physiological signals from movement artifacts.^[^ ^311^, ^312^ ^]^ Variability due to skin pigmentation is mitigated through the use of multi‐wavelength light sources, including near‐infrared LEDs, which offer more consistent penetration across skin types. Additionally, calibration algorithms and individualized baseline adjustments further enhance signal reliability across diverse users.^[^ ^313^, ^314^ ^]^

Implantable PPG sensors have also been explored for applications including muscle tissue monitoring. Biodegradability represents another promising future direction, as it eliminates the need for surgical removal. Researchers have already developed biodegradable optical filters for implantable applications, and Lu et al. introduced a biodegradable LED covering the PPG‐relevant wavelength range.^[^ ^315^ ^]^ With advancements in other functional components (e.g., photodiodes and batteries), a fully biodegradable PPG system appears increasingly promising.

### 5.2 Electrochemical Direct Muscle O_2_ Sensing

As an electroactive molecule, O_2_ undergoes cathodic reduction, generating a current proportional to its concentration. The measured current magnitude directly correlates with tissue oxygen tension or partial pressure levels (PtO_2_ or pO_2_). Structurally, electrochemical oxygen sensors typically consist of an Ag/AgCl reference electrode and an Au or Pt working electrode, both enclosed within a needle‐shaped sheath made of glass or stainless steel.^[^ ^316^ ^]^ A gas‐permeable membrane at the needle tip separates the liquid electrolyte from the target tissue, allowing selective diffusion of oxygen molecules. Alternative electrode materials, such as carbon‐based electrodes, have been investigated for their long‐term in vivo stability; however, they often cause significant tissue damage, making Pt electrodes the preferred choice.^[^ ^317^, ^318^ ^]^ Due to their invasive nature, these sensors are primarily used in clinical settings for monitoring muscle tissue oxygenation.^[^ ^319^ ^]^ One major drawback is the inherent time delay associated with single‐point measurements, which can lead to inaccuracies and potential misdiagnoses. Probe‐style Clark electrodes partially address this issue by enabling real‐time data acquisition.^[^ ^320^ ^]^ Nonetheless, precise calibration remains essential for accurate readings across all Clark electrode variants, as environmental factors, such as temperature and ionic strength, significantly influence sensor performance.^[^ ^316^ ^]^ Moreover, oxygen consumption by the probe during measurement presents challenges for repeated readings at a single site. Recent advances in microelectrode design have mitigated some of these limitations by reducing tissue damage and enhancing spatial resolution during in vivo recordings.^[^ ^321^, ^322^ ^]^

Alternatively, transcutaneous oxygen tension monitoring (TcpO_2_ or TCOM) offers a less invasive method compared to pO_2_ for measuring tissue oxygen levels at the skin surface. In addition, unlike PPG, which estimates oxygenation indirectly by measuring (de)oxygenated hemoglobin levels, TcpO_2_ directly measures tissue oxygenation levels (Figure 7b). TcpO_2_ read‐outs are either electrochemical with a planar Clark electrode (electrochemical TcpO_2_) or are optical using luminescence quenching interfaces (optical TcpO_2_). TcpO_2_ systems typically require heating elements to elevate the skin temperature to ≈45 °C, thereby enhancing blood flow, oxygen diffusion, and skin permeability.^[^ ^323^ ^]^ As a result, TcpO_2_ measurements remain underexplored in wearable technologies. Electrochemical TcpO_2_ encounters inherent limitations of Clark electrode O_2_ sensing, such as O_2_ consumption during monitoring, frequency calibration requirements, and susceptibility to motion artifacts during ambulatory TcpO_2_ measurements. Recent advances in skin‐like stretchable electronics present an opportunity to address some of these challenges.^[^ ^87^, ^324^, ^325^ ^]^ For example, electrochemical TcpO_2_ monitoring systems have been integrated into a wearable sensing patch.^[^ ^326^ ^]^ This adhesive‐free, stretchable patch incorporated a gold electrode coated with Nafion, featuring a selective diffusion membrane made of PDMS. However, its stability has been observed to last only for 30 s. Additionally, a Pt‐based stretchable and humidity‐insensitive O_2_ sensor utilizing a serpentine hydrogel fiber structure and a hydrophobic elastomer encapsulation was proposed, which eliminated corrosion and electrolyte consumption, thereby significantly prolonging the lifespan of the sensor.^[^ ^327^ ^]^ Advancement in Clark electrodes involves the incorporation of a 3‐electrode system, which includes a counter or auxiliary electrode to increase the stability and accuracy of measurements.^[^ ^328^ ^]^ More recent advancements have focused on modifications to the physical geometry of commonly used configurations.^[^ ^329^ ^]^ Few examples include the use of transient‐doped organic electrochemical transistors to enhance sensitivity and enable the detection of low O_2_ levels,^[^ ^330^ ^]^ miniaturization techniques,^[^ ^331^ ^]^ the incorporation of non‐noble metals, including membrane‐free electrodes like Pt micro‐discs,^[^ ^332^ ^]^ and metal‐polymer electrodes.^[^ ^333^ ^]^ Despite these improvements, challenges related to membrane manipulation and fragility persist.

Implantable Pt‐based electrochemical sensors have been reported to monitor continuously the peripheral muscle tissue O_2_. Rivas et al. proposed a Pt‐based micro‐needle wireless implantable electrochemical sensor for in vivo pO_2_ measurement in intramuscular tissue, although the device calibration remained the main limitation.^[^ ^334^ ^]^

Despite the significant biomedical and clinical importance of direct tissue oxygenation monitoring, effective electrochemical methods for non‐disruptive, fully non‐invasive, and continuous monitoring solutions are still lacking.^[^ ^335^, ^336^ ^]^ Electrochemical transcutaneous methods provide a non‐invasive alternative, though they require heating. However, for muscle oxygenation monitoring, non‐enzymatic (amperometric) electrochemical O_2_ sensors are the current gold standard for directly evaluating pO_2_.^[^ ^337^ ^]^ Therefore, current research may explore micro‐needle approaches for pO_2_ monitoring to improve real‐time accuracy, eliminate heating requirements, and enable minimally invasive continuous monitoring.

### 5.3 Luminescence‐based Direct Muscle O_2_ Sensing

A key advantage of luminescence‐based optical oxygen (O_2_) sensing over electrochemical methods is the absence of O_2_ consumption during measurement, ensuring that recordings more accurately reflect oxygen concentrations within muscle tissue. Additionally, optical TcpO_2_ monitoring has been reported to outperform electrochemical techniques in mitigating motion artifacts.^[^ ^338^, ^339^ ^]^

Luminescence‐based O_2_ sensors incorporate light emitters, detectors, and chemically immobilized oxygen‐sensitive molecules. Upon light excitation, these luminophores undergo luminescence quenching, a process in which excited‐state molecules transfer energy to nearby O_2_ molecules, causing emission decay. This quenching phenomenon affects recorded luminescence intensity and lifetime according to the Stern‐Volmer equation (Figure 7a), wherein both parameters are inversely proportional to the O_2_ concentration (Figure 7c). Intensity‐based measurements are relatively simple to implement but are often affected by non‐uniform illumination and uneven distribution of probe molecules.^[^ ^340^ ^]^ In contrast, lifetime‐based techniques are more robust, as they are largely independent of excitation intensity, detector sensitivity, and probe concentration. As a result, luminescence‐based sensors are increasingly favored for muscle oxygenation monitoring, especially given recent advances in miniaturized optics and detectors.^[^ ^341^ ^]^ These systems can be implemented using either time‐domain or frequency‐domain techniques. In time‐domain methods, probe molecules are excited with a short light pulse, and the resulting decay profile is captured using time‐correlated single‐photon counting or time‐gated imaging systems. In contrast, frequency‐domain techniques employ modulated light sources and determine emission lifetimes by measuring the phase shift between excitation and emission signals. Fluorescence quenching occurs when the fluorescence intensity of a fluorescent molecule decreases rapidly, on the order of ns to µs, upon interaction with a quencher molecule. In contrast, phosphorescence quenching involves a similar decrease in the phosphorescence intensity of a phosphorescent molecule but over longer timescales, from µs to ms.^[^ ^342^ ^]^ Fluorescence‐based sensing faces selectivity challenges due to spectral overlap with endogenous fluorophores such as melanin, collagen, and elastin.^[^ ^342^ ^]^ To overcome this, probes must be engineered with longer excitation and emission wavelengths to distinguish sensor signals from autofluorescence, or alternatively, rely on delayed fluorescence detection.^[^ ^343^ ^]^ Phosphorescent probes, with inherently longer emission lifetimes, enable more effective separation of target signals from background emissions. Consequently, most wearable and implantable O_2_ sensors for muscle oxygenation monitoring utilize lifetime‐based phosphorescence quenching, due to its ability to avoid interference from tissue autofluorescence and simplify gating requirements on the detector end.^[^ ^341^, ^342^ ^]^

In general, optical O_2_ sensors are positioned at the tips of optical fibers or coated on the surroundings to leverage waveguide evanescent fields (mainly to be used in the operation room) or embedded within soft polymeric films (to be used in wearable and implantable devices).^[^ ^344^ ^]^ Although optical fiber tip‐embedded systems are primarily utilized during surgeries, the evanescent field detection method also holds promise for the chip‐scale miniaturization of these sensors, facilitated by advancements in photonic integrated circuits. This topic has not yet attracted much research focus, which may change considering the advances in new materials such as lithium niobates.^[^ ^345^ ^]^ Nevertheless, soft materials embedded with O_2_‐sensitive dyes already show great potential for skin‐interfaced wearable devices such as smart patches, minimally invasive microneedle‐based systems, and injectable implant applications, as will be discussed in the following subsections.

#### 5.3.1 Wearables for Luminescence‐based Muscle O_2_ Monitoring

The development of oxygen‐sensitive materials is a critical aspect of tissue oxygenation monitoring technologies. The luminescence brightness of these sensing molecules directly enhances the system's SNR, contributing to improved detection sensitivity.^[^ ^346^ ^]^ Evans's group is at the forefront of synthesizing O_2_‐sensitive materials for wearables using the “click” chemistry, which emits a bright red light even visible to the naked eye.^[^ ^347^, ^348^ ^]^

Toward wearable system development, Evans's group first developed a portable fiber‐optic device used for diagnosing and treating acute compartment syndrome patients.^[^ ^349^ ^]^ The device features a tip coated with a poly(propyl methacrylate) (PPMA) matrix containing a Pt(II)‐core porphyrin. This porphyrin emits at λ = 645 nm when excited with blue light (λ = 377 nm) or green light (λ = 531 nm). Various materials, including tetraethoxysilane (TEOS) sol‐gel, PDMS, 3M Cavilon, and poly(ethyl methacrylate) (PEMA), were evaluated as matrix materials for hosting the porphyrin O_2_ sensor. Their in‐house synthesized Pt(II)‐pivaloyl porphyrin in PPMA demonstrated independence from humidity or pH and exhibited a low photobleaching rate, essential for precise intensity‐based pO_2_ measurements in intramuscular applications. In their later works, PPMA emerged as an interesting matrix material considering its highest sensitivity, rapid response time, excellent photostability, high brightness, mechanical flexibility, and resilience to humidity.^[^ ^348^ ^]^ While TEOS showed comparable response time and O_2_ sensitivity, its poor mechanical strength in support matrices restricted its use. Furthermore, the study highlighted that the minimal aggregation of pivaloyl porphyrin contributed to its compatibility with PPMA support matrices. The group later adopted a lifetime‐based method for measuring O_2_ concentration instead of intensity‐based phosphorescence quenching, which mitigated issues like photobleaching, enhanced signal, and motion stability.^[^ ^350^ ^]^ Although the lifetime signal exhibited a slightly lower overall O_2_ sensing resolution compared to intensity‐based devices, it was reported to satisfy the medical diagnostic requirements of the application and demonstrated greater robustness to temperature variations.^[^ ^350^ ^]^

In 2017, Park's research group developed the first wearable optical TcpO_2_ system, designed in the form of a flexible smart patch for the fingertip (Figure 7d). Their compact system featured a sensitive dye with porphyrin platinum(II), a 535 nm OLED, and an OPD as a light detector, resulting in a thin, imperceptible solution.^[^ ^351^ ^]^ Prior to these studies, in 2008, planar oxygen sensors were initially utilized in situ for clinical pO_2_ monitoring, rather than being specifically designed for wearable TcpO_2_ applications.^[^ ^352^ ^]^ TcpO_2_ monitoring is a non‐invasive technique that measures pO_2_ diffusion through the skin, closely correlating with tissue oxygenation and arterial oxygen levels.^[^ ^353^ ^]^ In a subsequent study, Park's group employed an indium tin oxide (ITO) thin‐film heater to increase skin temperature, thereby enhancing oxygen diffusion efficiency. This approach differed from their 2017 study, which sought to conduct wearable TcpO_2_ measurements without the use of a heating element. Furthermore, their more recent 2019 study improved light emission performance by replacing the OLED with a µLED, thereby enabling TcpO_2_ measurements over extended periods of up to 60 min, an achievement not previously possible with the ambient air‐unstable OLED.^[^ ^354^ ^]^ Later, Evans's group developed a wearable TcpO_2_ sensor that differed from standard systems by omitting heating elements and being entirely self‐contained (Figure 7e).^[^ ^354^ ^]^ Their O_2_‐sensitive materials with high brightness, based on phosphorescence quenching, enable TcpO_2_ quantification without the need for heating to enhance oxygen diffusion. This device integrates optics, electronic readout, and data processing capabilities into a compact, lightweight package weighing less than 30 grams. The system has been tested under various atmospheric conditions, including temperature and humidity fluctuations, demonstrating its reliability. The film structure included vertically stacked layers: a semi‐permeable transparent membrane isolating the skin from atmospheric O_2_, a PPMA film embedding metalloporphyrins, a transparent and breathable membrane, and a spin‐coated white breathable layer. This design enhanced emission collection through backscattering while providing optical insulation from external light sources. Despite initial success, the sensitivity of the device to O_2_ was initially constrained by hardware limitations. For instance, physical separation later mitigated the LED emission leakage into the photodiode signal using an opaque layer of black nail polish.^[^ ^355^ ^]^ Furthermore, enhanced calibration algorithms were developed for human pilot studies, integrating phosphorescence intensity and lifetime readings into a unified pO_2_ metric.^[^ ^355^ ^]^ These algorithms also enhanced the Stern–Volmer relation by incorporating temperature dependence and an explicit photobleaching term, ensuring readings independent of temperature changes and photobleaching.^[^ ^356^ ^]^

Marks et al. developed an intelligent dressing for chronic wounds in muscle vitality analysis by integrating O_2_‐sensitive material into a swellable hydrogel matrix.^[^ ^357^ ^]^ This dressing allows qualitative analysis of muscle O_2_ and pH levels by the naked eye and quantitative analysis using a camera. This functionality is enabled by a porphyrin conjugate combined with polyethylene glycol (PEG) amine polymer, which quickly crosslinks at room temperature, creating a color‐changing hydrogel dressing with adjustable swelling properties suitable for various wound environments. In addition, smart patches must balance breathability to prevent operator discomfort due to irritation and minimize the risk of redness or swelling upon removal.^[^ ^358^ ^]^ This permeability compromises TcpO2 measurement by allowing atmospheric O_2_ interference with tissue O2 diffusion. Therefore, TcpO2 devices should utilize semi‐breathable materials that facilitate partial moisture and O2 diffusion, ensuring accurate measurements that require precise calibration.^[^ ^359^ ^]^

Recognizing the influence of the skin barrier in limiting O_2_ exchange, which presents challenges in measuring rapidly fluctuating tissue O_2_ partial pressure, as well as the need for an extensive calibration algorithm fine‐tuning to correlate pO_2_ with TcpO_2_ values, researchers proposed an alternative solution for continuous wearable TcpO_2_ monitoring. Building on advancements in microneedle technology.^[^ ^360^, ^361^ ^]^ Müller et al. developed microneedles incorporating O_2_‐sensitive cyclohexenyl pivaloyl Pt‐core porphyrin (Figure 7f).^[^ ^362^ ^]^ For accurate operation, microneedles must be O_2_‐permeable, with a non‐permeable backing to prevent interference with the measurements. They should also be biocompatible, transparent for light transmission, and strong enough to penetrate the stratum corneum without breaking. Consequently, PEMA and PPMA were evaluated for their high O_2_ permeability and biocompatibility, while PLA was selected as the backing material due to its biocompatibility, transparency, and low O_2_ permeability. The group reported successful measurement of O_2_ underneath the skin at physiologically relevant pO_2_ levels (0–160 mmHg). The microneedle array consisted of 6 × 6 microneedles with a length and pitch of 1000 µm, designed to avoid triggering pain receptor neurons. Indeed, this device offered minimal invasiveness compared to TcpO_2_ systems but did not require the extensive calibration typically associated with them. On the other hand, it provided an alternative to implantable devices, which were known to be associated with over 50% of healthcare‐related infections.^[^ ^363^ ^]^ They also developed dressings to monitor muscle tissue oxygenation during transplants, crucial to maintaining cellular viability and function, preventing tissue hypoxia and necrosis, and improving transplant success rates and long‐term outcomes for recipients.^[^ ^364^ ^]^

#### 5.3.2 Implants for Luminescence‐based Muscle O_2_ Monitoring

Generally, O_2_‐sensing materials designed for implantable oxygenation monitoring must exhibit solubility in the relevant physiological matrix, low toxicity, high stability, and excellent photophysical properties, including high quantum yield and sensitivity to molecular O_2_.^[^ ^365^ ^]^ Sonmezoglu et al. developed a miniaturized (4.5 mm^3^) implant for direct deep‐tissue pO_2_ monitoring, capable of providing continuous real‐time data from depths of several centimeters in sheep and up to 10 cm in *ex vivo* porcine muscle tissue (Figure 7g).^[^ ^366^ ^]^ Their device represented a fully integrated, wireless, ultrasound‐powered platform that utilized a luminescence lifetime of O_2_‐sensitive tris‐(bathophenanthroline) ruthenium (II) perchlorate dye to quantify muscle tissue O_2_ concentration. The system has a transceiver for bidirectional data transfer embedded in itself as well as a blue µLED, an optical filter for selective recording with an on‐chip photodetector, and integrated electronics for system operation. Although the current iteration of the device required surgical placement, future miniaturization efforts will enable semi‐invasive or vascular approaches for probe placement. Similarly, Cai et al. developed a comparable device that utilized wireless power transmission.^[^ ^367^ ^]^ Unlike Sonmezoglu's system, their approach relied on off‐the‐shelf components for electronics (Figure 7h). Nevertheless, they successfully developed a compact unit suitable for implantation, capable of real‐time continuous pO_2_ monitoring, which was validated in freely moving rodents.

Passive injectable transient implants offer a simpler alternative to traditional implants, allowing delivery through minimally invasive surgery, natural biodegradation, and no need for follow‐up removal surgery. As their optical readout remains external, these implants must use excitation sources aligned with the tissue optical window to enable deep light penetration and minimize signal loss. To meet these criteria, Chimene et al. developed O_2_‐sensing devices injectable via 18‐gauge needles by embedding phosphors such as palladium(II) meso‐tetra‐(sulfophenyl) tetrabenzoporphyrin sodium salt and PdBP into hydrogel matrices, while Falcucci et al. created a physically crosslinked silk protein network incorporating the O_2_‐sensitive chromophore PdBMAP.^[^ ^368^, ^369^ ^]^ These chromophores differ from traditional porphyrin‐based variants by exhibiting excitation at 630 nm and emission ≈800 nm, aligning with the tissue optical window (600–1300 nm) to enhance light penetration. A range of biodegradable host materials have been explored for integrating these O_2_‐sensitive chromophores to reduce long‐term forging body response, including synthetic polymers such as (2‐hydroxyethyl methacrylate) (poly‐HEMA),^[^ ^370^, ^371^ ^]^ PLA,^[^ ^372^ ^]^ and polycaprolactone (PCL)^[^ ^373^ ^]^ as well as natural polymers like gelatin and hyaluronic acid (HA).^[^ ^374^, ^375^ ^]^ While gelatin and HA offer excellent biocompatibility, their rapid degradation and limited mechanical integrity in vivo pose challenges. To address this, blending PCL with gelatin has been shown to mitigate these limitations by improving structural stability and extending degradation time.^[^ ^373^ ^]^ Leveraging the green synthesis advantages of silk fibroins, Presley et al. addressed chromophore aggregation by processing silk‐PdBMAP composites with 1,1,1,3,3,3‐hexafluoro‐2‐propanol to enhance homogeneity, while optimizing crystallinity, mechanical properties, and porosity to improve tissue integration and accuracy in muscle oxygenation monitoring.^[^ ^376^ ^]^

Advancements in tissue oxygenation monitoring are accelerating at a remarkable pace, with a strong emphasis on enhancing precision, user‐friendliness, and the integration of these technologies into medical wearables and implants. At the forefront are non‐invasive, real‐time luminescence‐based sensors, which are becoming more accurate, efficient, and accessible for everyday use. Researchers are pushing the boundaries by embedding these sensors into form factors like smart patches, microneedles, and implantable devices, all while improving both usability and signal accuracy. The incorporation of biocompatible materials is boosting sensor performance and longevity, while biodegradable, minimally invasive implants are overcoming the challenges of traditional device removal, particularly for temporary implants used to monitor localized variations post‐surgery. Furthermore, researchers are refining calibration algorithms to overcome motion artifacts, environmental changes, and skin tone variations, ensuring accurate and reliable measurements in real‐world conditions. As these technologies continue to evolve, they are set to become a cornerstone of personalized healthcare, revolutionizing chronic disease management, wound healing, and clinical diagnostics, harnessing the vital role tissue oxygenation plays in bodily functions.

## 6 Conclusion and Discussion

The growing adoption of wearable and implantable devices, coupled with a systemic shift in healthcare from reactive treatment to proactive, preventive care, has driven a major transformation in medical technologies. This shift is further propelled by the increasing demand for continuous and remote health monitoring to reduce hospitalization burdens and enable earlier diagnosis and more personalized intervention strategies. Technologically, these evolving clinical and societal demands are compelling the biomedical field to repurpose and refine microengineering techniques originally developed for integrated circuits. Specifically, the precision, scalability, and miniaturization achievements of the semiconductor industry, driven largely by CMOS technology, are now being adapted for use with soft, stretchable, and biocompatible materials. This convergence of microfabrication with material science is facilitating the development of conformal, low‐power, and multifunctional devices capable of seamless integration with the human body. Importantly, this represents not just a trend in miniaturization but a fundamental shift in how medical devices are conceptualized, designed, and deployed in real‐world clinical and home settings.

Muscle tissues are central to vital functions, from movement and cardiac activity to organ contractions, making real‐time muscle signal monitoring essential for medical diagnostics, rehabilitation, human‐machine interfaces, robotic control, and even virtual reality‐based human augmentation. Transitioning healthcare from a reactive to a proactive model demands significant technological advancements in continuous physiological monitoring. For these tools to be effective, they must deliver precise physiological insights while remaining user‐friendly and unobtrusive. Advancements in material science and structural design are key to overcoming challenges in continuous muscle bio‐signal monitoring, with new devices emerging daily to enhance sensing performance. In this review, we have systematically examined the latest progress in miniaturized wearable and implantable devices for continuous muscle electrophysiology, biomechanics, and oxygenation monitoring. We illustrated ten representative muscle monitoring technologies, providing brief introductions to their operational mechanisms. We focused this review on materials and device architectures that boost sensing performance in both wearable and implantable formats and left out topics such as circuitry innovations, wireless communication, power management, data acquisition, and processing algorithms.

Advances in muscle sensing technologies have led to a diverse landscape of wearable and implantable sensors. Electrophysiological modalities such as ECG and EMG both offer high temporal resolution and direct neural interfacing. EMG, in particular, provides finer spatial resolution than ECG as it is capable of capturing individual motor unit activities with intramuscular electrodes. Although wearable ECG and EMG systems share similar fundamental requirements, the higher signal frequency and greater mechanical deformation associated with EMG impose more stringent demands on material properties and device design. On the other hand, MMG, despite its advantages of being contactless, offering higher spatial resolution, and being immune to electrode‐skin impedance, is limited by its inherently low signal amplitudes and the need for complex magnetic shielding. These challenges have hindered its widespread adoption compared to ECG and EMG. Biomechanical approaches such as FMG, AMG, SMG, and EIM offer complementary insights by detecting mechanical, acoustic, and impedance changes during muscle contraction. FMG excels in wearability and robustness but suffers from lower spatial specificity. With advancements in new energy harvesting materials, future FMG systems hold promising potential for self‐powered operation. SMG and AMG, based on ultrasound and acoustic waves, respectively, are promising for dynamic muscle detection. Compared to the passive detection nature of AMG, most SMG systems remain relatively bulky or power‐intensive, particularly in continuous wearable applications. However, due to the stronger signal intensity, 2D imaging capability, and greater muscle penetration depth of SMG, it continues to attract active research and development efforts. EIM provides volumetric and tissue property data, but is highly sensitive to electrode placement. Beyond active contractions detection, the impedance measured in EIM also reflects the structural and compositional properties of muscle tissue. For tissue oxygenation, PPG oxygen sensors are dominant in non‐invasive systems, offering heart rate and oxygen saturation data through optoelectronic components. However, the wearable format is limited by shallow penetration depth. Implantable PPG systems offer a larger illumination area, reduced light attenuation, and less noise/artifacts from ambient light, thereby enhancing signal strength and overall sensing performance. Electrochemical and luminescence‐based oxygen sensors offer direct measurement of tissue oxygenation, particularly in their implantable forms. While luminescence‐based methods have gained significant attention in recent years—showing strong technological advancements for deep‐tissue and organ‐level monitoring—they still face challenges related to long‐term biocompatibility and stability. Electrochemical sensors, on the other hand, struggle with calibration issues in wearable formats, making consistent and reliable measurements more difficult outside of controlled settings (**Table** **5**).

**Table 5:** summarizes the key technology advantages, limitations, and representative milestones of each sensing modality over the past decade.

Technology	Wearable Advantage	Wearable Drawbacks	Implant Advantage	Implant Drawbacks	Milestones	Refs.
EC G	Low‐noise and low power detection Simple integration in	Limited spatial resolution	Stable signal fidelity Capability of ECG mapping with high SNR	Invasive	First ECG wearable sensor with true 3D integration	Huang *et al.* in 2018.[85]
EMG	High temporal resolution Simple integration in daily wearables	Adjacent muscles crosstalk Susceptible to motion and skin impedance	High SNR Single motor unit resolution Deep muscle access	Complex signal processing for high‐ density signal decomposition	iEMG sensor for decomposition of motor unit activity	Muceli *et al.* 2022.[156]
MMG	High spatial resolution	Vulnerability to ambient magnetic noise Weak signals	Improved SNR and fidelity from deep muscle Better biocompatibility	Invasive Few use cases	First miniaturized MMG system using TMR sensors that achieves muscle magnetic signal detection at RT	Zuo *et al.* in 2020. [172]
FMG	Insensitive to electrical noise Low power	Limited spatial resolution Affected by sensor placement and tightness	Direct muscle force monitoring Robust sensing, as no electrical interface with tissue	Mechanical signal drift due to fibrotic tissue growth.	ID capacitive strain sensor with a sensitivity (—12) three times higher than the highest existing one	Lee *et al.* 2021.[218]
EIM	Sensitive to muscle structure and composition Low‐cost electronics	Sensitivity to electrode placement and motion	Precise impedance tracking Improved localization and signal fidelit	Few devices exist Tissue heterogeneity effects	First dynamic EIT‐based fabric sensor with deep learning correction for real‐time pressure mapping	Duan *et al.* 2019. [378]
AMG	Non‐invasive Robust to sensor placement variability	Limited to surface muscles Vulnerable to environmental sound noise	NA	NA	First soft sensor platform capable of AMG together with ECG, speech, and LVAD monitorinu	Liu *et al.* I 2016. [251]
SMG	Non‐invasive Deep detection High spatial resolution	Limited temporal resolution Relatively complex structure	NA	NA	First truly skin‐conformable SMG device capable of continuous deep cardiac muscle tissue imaging	Hu *et al.* in 2023. [256]
PPG	High comfort and easy Integration Continuous pulse light tracking	Shallow Penetration Prone to noise/artifacts from ambient light	Deep muscle accessibility. Larger tissue detection volume and enhanced signal sensitivity	Implant heating Limited battery lifetime	First demonstration of a flexible, printed organic PPG array for 2D oxygenation mapping	Khan *et al.* in 2018.[287]
Electroch emical	non‐invasive	Calibration complexity	Minimal interference from motion High SNR Less calibration requirements	Biofouling over time Limited long‐term stability	Micro‐needle wireless implantable electrochemical sensor for in vivo p02 measurement	Rivas *et al.* in 2020.[334]
Lu mínese ence‐based	non‐invasive Simple integration in daily wearables	Calibration complexity	Minimal interference from motion High SNR Less calibration requirements	Biofouling over time Limited long‐term stability	First optical wireless wearable system for non‐invasive Tcp02 monitoring.	Cáscales *el al*. in 2020.[354]

For wearable devices, key considerations include ensuring permeability and breathability to minimize skin irritation, achieving a high SNR for precise signal acquisition, maintaining stable attachment with self‐adhesive properties for prolonged use, and utilizing flexible, lightweight, and biocompatible materials to enhance user comfort and safety. Implantable devices demand additional attention, such as minimizing mechanical mismatches with tissue, mitigating cytotoxicity risks, and meeting stringent biocompatibility standards. They also prioritize ultra‐low power operation and wireless power and data transfer, ideally functioning autonomously without external power sources or cumbersome wiring. Each muscle‐monitoring modality presents unique technical challenges: ECG and EMG require conformal skin contact to minimize interface impedance; FMG focuses on achieving high linearity and sensitivity; and luminescence‐based oxygen sensors demand exceptional photophysical performance for efficient light penetration. There is also a search for materials and architectures to improve stretchability, integration density, and functional complexity for the muscular sensing system.

Despite advances in muscular sensing, key challenges persist. Looking ahead, future research should focus on integrating multimodal sensing, combining electrophysiological, biomechanical, and biochemical signals to provide a holistic view of muscle status. These integrations require the development of new materials with tailored mechanical‐electrical‐biochemical properties. Overlapping sensor responses may cause interference, making clinical validation crucial for reliability. Besides, integrating diverse sensing mechanisms can also offset individual limitations and create synergistic benefits.

System‐level integration in muscular sensors has advanced in power methods, including wireless harvesting via coils and bioenergy conversion using piezoelectric and mechanoelectrical materials. Wireless communication (BLE, ultrasound, infrared light) enables untethered data transfer. While muscular biosignals are widely utilized in HMI and robotic control, achieving high signal interpretation accuracy without increasing latency remains challenging. High‐density sensing arrays can increase accuracy, but they also generate large data streams, leading to longer processing times. The emerging field of data compression and edge artificial intelligence in CMOS technology will be essential to achieve rapid and precise data extraction and processing at the edge. Recent developments in non‐volatile memory, such as memristors, ultra‐low‐voltage 2D‐material resistive memories, and van der Waals flash memory devices, could offer compelling on‐device storage solutions for future muscular biosensing platforms. To sum up, current bottlenecks include limited power, constrained edge computing capacity, and unstable wireless links due to tissue attenuation and motion artifacts. Promising solutions involve bioenergy harvesting, miniaturized edge CMOS, and multimodal communications for greater reliability in biological environments.

Biocompatibility, biodegradability, and long‐term signal stability are essential considerations for the safe and reliable operation of implantable muscular sensing systems. Some reviewed studies have reported >90% cell viability in vitro and minimal immune response or fibrosis in long‐term in vivo tests. However, challenges such as chronic inflammation and fibrotic encapsulation, particularly the formation of thick fibrotic layers, can lead to progressive signal degradation over time. Future strategies such as anti‐inflammatory coatings, advanced fixation, and precise material engineering to match tissue properties are essential. Biodegradable materials offer a promising implant route, eliminating the need for surgical retrieval. However, current limitations in biodegradable sensors stem from the lack of materials that can balance biocompatibility, suitable functional properties, and well‐controlled degradation profiles. Materials often degrade too quickly or too slowly, leading to premature sensor failure or prolonged foreign‐body response. Beyond precise material engineering, future research may explore tunable hybrid biodegradable materials with controlled properties. Current implantable muscular sensing systems have shown certain stability across diverse technologies, maintaining functionality under strain, bending, and extended immersion in physiological environments. Future efforts may focus on developing anti‐fouling surfaces, adaptive calibration strategies, and real‐time signal correction to maintain sensing fidelity over extended periods.

Current studies largely focus on skeletal and cardiac muscle, with smooth muscle, critical for gastrointestinal and organ function, often overlooked. Real‐time monitoring of smooth muscle activity can become a reality by including muscular sensors in ingestible devices. Such technologies could be used to track digestive system functions or assess post‐transplant conditions. Also, spatial coverage in muscle sensing remains limited, yet expanding sensing areas could enable more accurate movement decoding and disease localization. The challenge is that large covering areas limit the sweat and heat dissipation functions of the skin. Intrinsically soft materials are gaining attention due to their superior flexibility and conformability. Future research could further explore elastic modulus gradients at rigid‐soft boundaries and develop skin‐mimicking interfaces with properties like self‐healing, durability, and thermal sensitivity.

We envision that the integration of novel materials, innovative structural designs, and scalable fabrication techniques will drive the next generation of muscular biosignal monitoring systems that are accurate, comfortable, and robust. These breakthrough platforms will lay the foundation for future wearable and implantable medical devices. Beyond diagnostics and therapeutics, they will play a pivotal role in rehabilitation, human–machine interfaces, and digital health. Ultimately, such systems will unlock transformative opportunities in personalized, preventive healthcare, paving the way for smarter, more adaptive, and patient‐centered medical care.

## Conflict of Interest

The authors declare no conflict of interest.
